# Membrane protein CRISPR screen identifies RPSA as an essential host factor for porcine epidemic diarrhea virus replication

**DOI:** 10.1128/jvi.00649-25

**Published:** 2025-07-30

**Authors:** Yu Zhao, Guanghao Guo, Yumei Sun, Mengjia Zhang, Gan Yang, Zhongzhu Liu, Yanbin Song, Ahmed H. Ghonaim, Ningning Ma, Mengdi Zhang, Anan Jongkaewwattana, Qigai He, Wentao Li

**Affiliations:** 1 National Key Laboratory of Agricultural Microbiology, Hubei Hongshan Laboratory, College of Veterinary Medicine, Huazhong Agricultural University 627716, Wuhan, China; 2 Virology and Cell Technology Research Team, National Center for Genetic Engineering and Biotechnology (BIOTEC), National Science and Technology Development Agency (NSTDA) 67960, Khlong Nueng, Pathum Thani, Thailand; University of Kentucky College of Medicine, Lexington, Kentucky, USA

**Keywords:** Porcine epidemic diarrhea virus, CRISPR/Cas9, RPSA, ERK1/2 pathway, lipid metabolism, aminopeptidase N

## Abstract

Porcine epidemic diarrhea, caused by porcine epidemic diarrhea virus (PEDV), is one of the most devastating diseases in the global pig industry due to its high mortality rate in piglets. The host factors required for PEDV replication, including receptors, remain poorly understood. Here, we developed a porcine membrane-protein-scale CRISPR/Cas9 knockout (PigMpCKO) library and performed two rounds of PEDV infection. Ribosomal protein SA (RPSA), the known receptor of dengue virus, was found to be a potent host factor. Moreover, our research revealed that RPSA is involved in the replication stage of PEDV and not in the entry stage. Inhibitor and activator experiments demonstrated that knockout (KO) of RPSA downregulates the ERK1/2 signaling pathway to impair PEDV infection. Additionally, RNA sequencing data indicated that cellular lipid biosynthesis and lipid transport processes were significantly inhibited in the absence of RPSA during PEDV infection. Mechanistic studies revealed that the reduction in total cholesterol and triglyceride levels, resulting from RPSA KO, was partially mediated by the ERK1/2 pathway, leading to impaired lipid accumulation during PEDV replication. Interestingly, RPSA KO also significantly downregulated the expression of aminopeptidase N (APN) and inhibited infection by transmissible gastroenteritis virus (TGEV) and porcine deltacoronavirus (PDCoV), both of which belong to the swine enteric coronavirus group. In summary, our results establish RPSA as a novel host factor that is critical for coronavirus replication. This provides new insights into the mechanisms of virus-host interactions and paves the way for the development of broad-spectrum antiviral therapies.

**IMPORTANCE:**

Swine enteric coronaviruses (SeCoVs) cause severe economic losses to the global swine industry and pose a potential threat to public health. Identification of receptors required for PEDV infection could develop novel targets for drug therapy and disease-resistant breeding. We conducted a CRISPR/Cas9 screen targeting membrane proteins in porcine kidney cells infected with PEDV to identify possible receptors and discovered numerous novel candidate host factors. Considering RPSA’s known role as a receptor for multiple viruses, we focused on investigating its potential in coronavirus infection. Our results revealed that RPSA does not contribute to the entry stage but to the replication stage of coronavirus infection. We first reported the role that RPSA plays in the regulation of APN expression and lipid metabolism. RPSA is essential for PEDV and other SeCoVs replication, providing a novel insight into the search for the receptor of PEDV and identifying potential therapeutic targets for coronaviruses.

## INTRODUCTION

Porcine epidemic diarrhea virus (PEDV), a member of the genus Alphacoronavirus, causes acute diarrhea, vomiting, dehydration, and high mortality in neonatal piglets (1). Since its emergence in the UK in the early 1970s, PEDV has led to both epidemic and endemic infections in pig populations worldwide and has significantly hampered the development of the global swine industry (2, 3). Unfortunately, most commercial vaccines are insufficient to provide effective protection against the prevalent PEDV mutant strains of the GII genotype (4, 5). Although the host factors involved in PEDV-host interactions are important targets for drug development and gene-editing breeding, they remain unclear, which hinders the development of countermeasures against PED (6). Therefore, a comprehensive understanding of the host factors and pathways co-opted by PEDV is urgently needed for the development of effective therapies to treat PED and prevent potential future outbreaks.

Recent studies employing CRISPR/Cas9 screening have addressed significant gaps in our understanding of the mechanisms of virus-host interactions and have successfully identified host genes essential for infection of several viruses, including SARS-CoV-2, Japanese encephalitis virus (JEV), and PDCoV (7–10). An earlier genome-wide CRISPRa screen identified the paired immunoglobulin-like receptor B (PirB) as a receptor for mammalian orthoreovirus (11). While receptor binding and cell entry are critical steps in the viral infection cycle, the functional receptor for PEDV has not yet been identified although it was discovered over 40 years ago (12). The role of APN as a receptor for PEDV remains controversial. Some studies suggest that APN may serve as a cell surface receptor for PEDV (13, 14), while recent evidence suggests that APN is not essential for PEDV entry, as APN knockout pigs remain susceptible to PEDV infection (15–17). To explore host factors, particularly potential receptors crucial for PEDV infection, we executed a membrane-protein CRISPR screen using LLC-PK1 cells.

Ribosomal protein SA (RPSA), also known as laminin receptor 1, is ubiquitously expressed and plays a multifunctional role in various cellular processes, including laminin binding, rRNA processing, and regulation of proinflammatory cytokines (18, 19). Additionally, RPSA negatively regulates MAPK pathway activation during foot-and-mouth disease virus (FMDV) infection, resulting in antiviral activity (20). Previous studies have demonstrated that PEDV infection in Vero cells activates three classical MAPK cascades: extracellular signal-regulated kinase (ERK), p38, and c-Jun NH2-terminal kinase (JNK) (21, 22). However, it remains unclear whether RPSA regulates PEDV replication via the MAPK pathway. Lipids, as major components and structural elements of enveloped viruses, are manipulated at various stages of the viral life cycle to promote viral replication (23, 24). For example, PEDV enhances the transcriptional activity of sterol regulatory element-binding transcription factor 1 (SREBF1) to increase cellular lipid accumulation (25), while inhibition of fatty acid β-oxidation reduces PEDV infection (26). However, the exact mechanism by which PEDV infection affects cellular lipid metabolism to facilitate replication requires further investigation.

In this study, we performed a CRISPR/Cas9 screen for membrane proteins to identify critical host factors, particularly potential receptors, involved in PEDV infection in LLC-PK1 cells. Among the top-scoring host factors, we focused on RPSA, which was found to be essential for PEDV infection, although it does not function as a receptor. Further experiments revealed that knockout of RPSA severely impaired the replication stage of the virus, with a decrease in viral particle production, which correlated with lower lipid droplet formation. We also demonstrated that RPSA positively regulates the ERK1/2 pathway and that RPSA KO inhibits PEDV replication by blocking ERK1/2 signaling in ST cells. Additionally, RPSA KO impaired cellular lipid synthesis and transport induced by PEDV infection, as determined by RNA-seq analysis and lipid contents tests. RPSA KO also inhibited PDCoV and TGEV infection, likely by suppressing ERK1/2 signaling and downregulating the expression of APN. In conclusion, our study suggests that RPSA represents a promising broad-spectrum target against porcine enteric coronaviruses.

## RESULTS

### CRISPR knockout library screening of membrane proteins identifies potential receptors for PEDV infection

**Fig 1:**
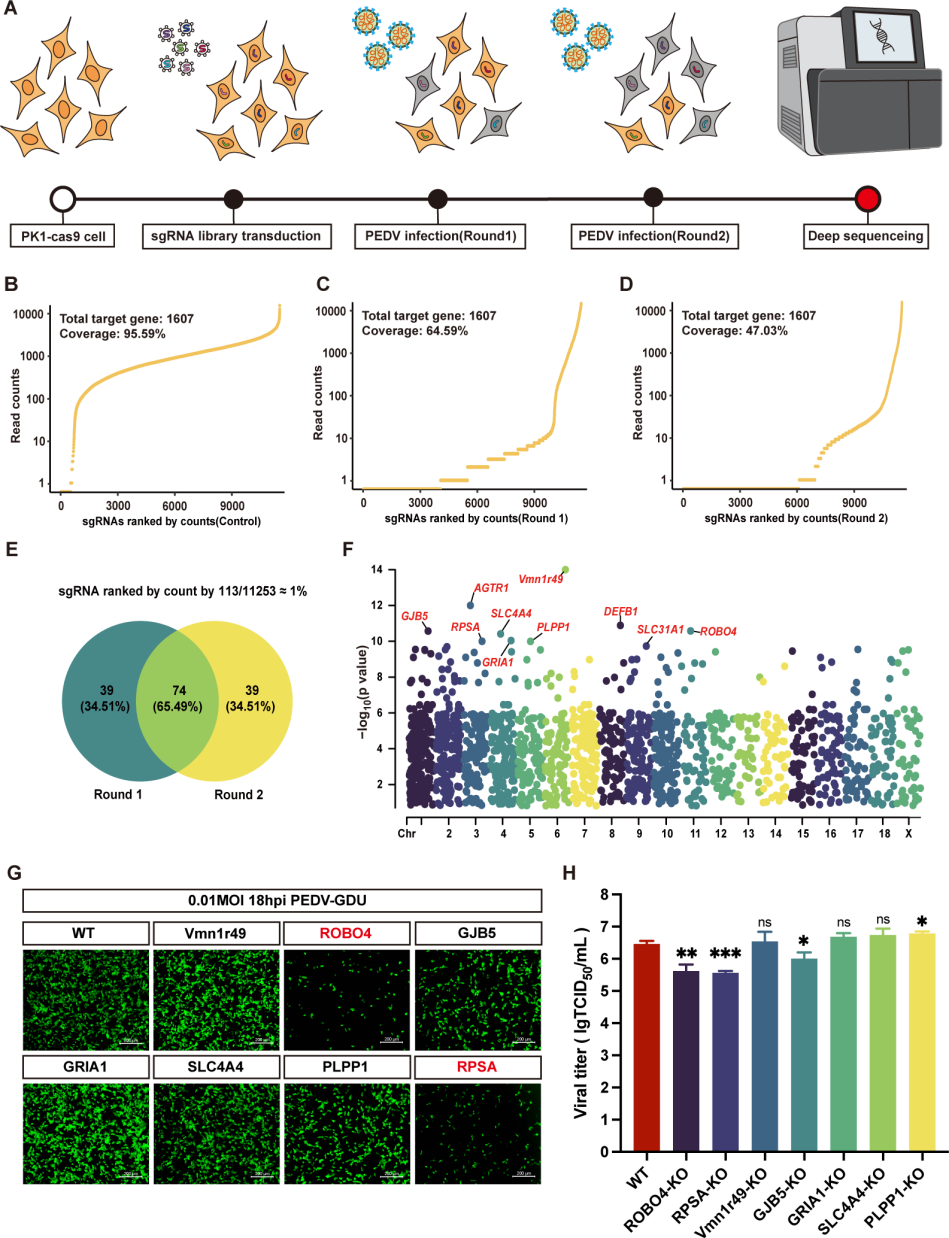
Membrane protein CRISPR screening to identify critical host factors associated with PEDV infection. (**A**) Membrane protein CRISPR screening workflow. Cas9-expressing LLC-PK1 cells were transduced with the membrane protein CRISPR lentivirus and subjected to two rounds of PEDV-GDU-GFP infection. Surviving cells and mock controls from each round of virus challenge were harvested for next-generation sequencing to assess sgRNA abundance. (**B–D**) Sequencing results of sgRNAs targeting sequences in the PigMpCKO cell library (**B**) and virus-challenged LLC-PK1-cas9 cell library after first round of infection (**C**) and second round of infection (**D**). The curve represents the distribution of sgRNAs. (**E**) Venn diagram showing the overlap of enriched sgRNAs among the top 1% of sgRNA reads from both PEDV screening rounds. (**F**) Manhattan plot highlighting the top 10 enriched genes identified in the second-round screen, with gene names labeled. (**G**) Fluorescence intensity in WT cells and seven selected gene knockout cells following infection with PEDV-GDU-GFP (MOI of 0.01) at 18 hpi. Scale bar, 200 µm. (**H**) Independent CRISPR knockouts were generated for the seven candidate genes, which were infected with PEDV-GDU-GFP at MOI of 0.01. At 12 hpi, viral infectivity was assessed by TCID_50_ assay. The values represent the viral titer (mean ± SD) from three independent experiments. ns, not significant; *P < 0.05; **P < 0.01; ***P < 0.001.

To identify host factors encoding potential receptors involved in PEDV infection, we constructed a PigMpCKO library in the LLC-PK1 cell line (Fig. 1A). This library consists of 11,052 sgRNAs targeting 1,607 membrane-protein-related genes and 200 non-targeted control sgRNAs, amounting to 11,252 sgRNAs in total, with a maximum of 7 sgRNAs designed per gene. Next, we introduced the sgRNA pool into LLC-PK1-Cas9 cells via lentiviral transduction at a multiplicity of infection (MOI) of 0.3. To determine the abundance of sgRNAs in the PK1-PigMpCKO mutant cell pool, we amplified the integrated sgRNA cassettes from genomic DNA by PCR and subjected them to Illumina sequencing. The sequencing results revealed that 95.59% (10,756 out of 11,252) of the originally designed sgRNA sequences were retained in the PK1-PigMpCKO cell collection (Fig. 1B).

We subsequently performed two consecutive rounds of independent CRISPR screens using the PEDV-GDU-GFP strain (GII-genotype) at an MOI of 1. Genomic DNA was extracted from the surviving cells, and the abundance of sgRNAs was determined by PCR and Illumina sequencing. The sequencing results revealed that 64.59% (7,236 out of 11,203) of sgRNAs were detected in the surviving cells after the first round of PEDV challenge, while 47.03% (5,276 out of 11,218) of sgRNAs were detected in the second round (Fig. 1C and D). Among the top 1% of the enriched sgRNAs (113 sgRNAs), 65.49% were co-enriched in both rounds of screening (Fig. 1E). The top 10 genes were identified by calculating the *P*-value of sgRNA differences between virus-treated and control samples in the second round of screening (Fig. 1F). Seven enrichment candidates were randomly selected for validation. Compared with wild-type STCas9 cells, knockout of ROBO4 or RPSA significantly decreased the viral titer, while knockout of other candidate genes did not affect viral replication (Fig. 1G and H). With these results, CRISPR/Cas9 screening identified several candidate membrane proteins involved in PEDV replication.

### RPSA is an essential host factor for PEDV infection but does not function as a receptor

**Fig 2:**
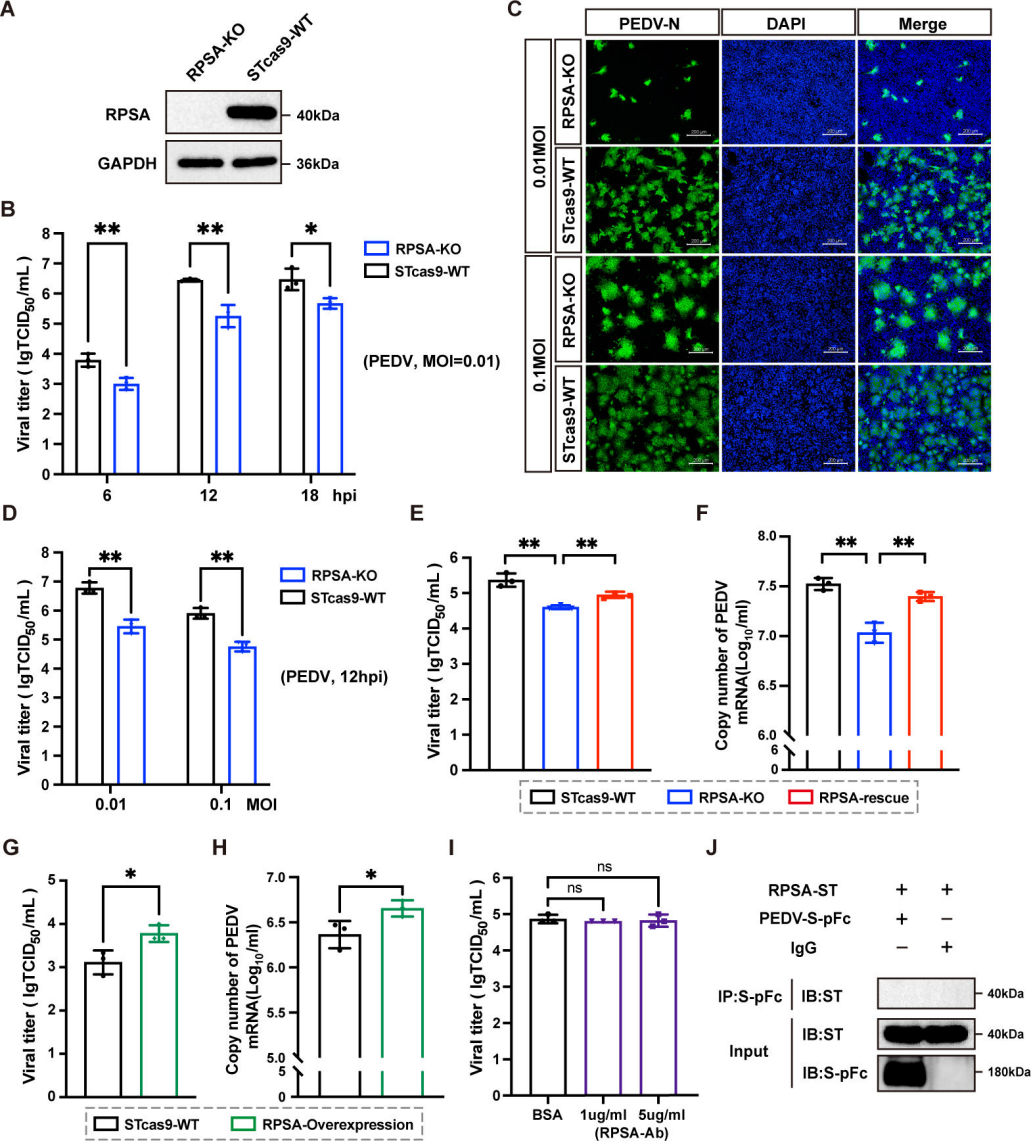
RPSA is a host factor required for PEDV replication. (**A**) Western blot analysis of endogenous RPSA protein expression in RPSA-KO cells and WT cells. GAPDH was used as the internal control. (**B**) RPSA-KO cells and WT cells were infected with PEDV-YN144 (MOI of 0.01) for different time points (6, 12, and 18 hpi). The viral titers were monitored by TCID_50_ assay. (**C and D**) RPSA-KO and WT cells were infected with PEDV-YN144 at different MOIs (0.01 and 0.1). PEDV N protein expression was detected via immunofluorescence assay at 12 hpi (**C**). Scale bar, 200 µm. PEDV titers (**D**) were tested at 12 hpi. (**E and F**) Rescue assays were performed in WT, RPSA-KO, and RPSA-rescue cells infected with PEDV-YN144 (MOI of 0.01) at 12 hpi. The PEDV titer was determined by TCID_50_ assay (**E**), and the PEDV N copy number was assessed via quantitative real-time PCR (**F**). (**G and H**) RPSA-overexpressing and WT cells following infection with PEDV-YN144 (MOI = 0.01) at 12 hpi. PEDV titers were assessed using TCID_50_ assay (**G**), and PEDV N gene expression was measured by RT-qPCR (**H**). (**I**) ST cells were incubated with 5 µg/mL BSA or 1 or 5 µg/mL antibodies targeting the C-terminus of RPSA for 2 h and then infected with PEDV-YN144 (MOI of 0.01) for 12 h. PEDV levels were measured by TCID_50_ assay. (**J**) Co-IP analysis to assess PEDV S protein binding to RPSA. HEK293T cells were transfected with pcDNA3.1-RPSA-ST for 24 h and then lysed. The cell lysates were incubated with Fc-Tagged PEDV S protein and then precipitated with protein A-coupled agarose beads. Co-purification of RPSA-ST was detected by Western blotting using anti-ST-Tag antibody. The means and SDs for the results from three independent experiments are shown. ns, not significant, *P < 0.05, **P < 0.01.

Previous studies have shown that RPSA serves as a functional receptor for several viruses (27–29). Therefore, we selected RPSA as a subject for further investigation. Additionally, our previous experiments demonstrated that ST cells are more susceptible to PEDV infection compared to LLC-PK1 cells. To evaluate the potential role of RPSA in PEDV infection, we generated an RPSA-KO STCas9 cell line via the CRISPR/Cas9 gene editing system. Successful construction of the RPSA-KO cell line was confirmed by Western blotting (Fig. 2A). Sanger sequencing also showed that the genome of the RPSA group had several base insertions and a fragment deletion compared to the WT group (Fig. S1A). Additionally, no significant differences in cell proliferation were observed between the clonal RPSA-KO cells and the wild-type (WT) cells, as assessed by a cell proliferation assay (CCK-8) (Fig. S1B).

Viral titration revealed significantly lower viral loads in RPSA-KO cells compared to WT cells at an MOI of 0.01, measured at 6, 12, and 18 h post-infection (hpi) (Fig. 2B). Indirect immunofluorescence analysis further revealed significantly reduced levels of PEDV N protein in RPSA-KO cells after infection at MOIs of 0.01 and 0.1 at 12 hpi (Fig. 2C). Consistent with these findings, viral titer assays performed at different MOIs also revealed significantly lower viral loads in RPSA-KO cells compared to WT cells (Fig. 2D). These results suggest that RPSA knockout significantly inhibits PEDV infection. To further confirm whether RPSA is essential for PEDV infection, we constructed an RPSA-KO-rescue cell line through lentiviral-mediated transduction (Fig. S1B and C). As shown in Fig. 2E and F, complementation of RPSA in RPSA-KO cells partially restored PEDV replication. Moreover, overexpression of RPSA in WT cells also enhanced PEDV replication (Fig. 2G and H; Fig. S1F). Collectively, these results indicate that RPSA is an essential host factor for PEDV infection.

Since RPSA spans the plasma membrane once, with its C-terminus exposed to the extracellular space (30), we performed an antibody inhibition assay to explore whether RPSA could act as a receptor for PEDV. STCas9 cells were pre-incubated with an antibody against the C-terminus of RPSA, which was confirmed by cell surface staining (Fig. S1G), and then infected with PEDV. The results showed that the antibody had no inhibitory effect on PEDV infection at concentrations of 1 or 5 µg/mL (Fig. 2I). Additionally, we overexpressed RPSA in PK15 cells, which are not susceptible to PEDV infection. The PK15 cell line overexpressing RPSA was successfully constructed and subsequently infected with PEDV for 12 h. Immunofluorescence analysis showed that the overexpression of RPSA did not initiate virus replication in PK15 cells (Fig. S1H). Co-immunoprecipitation assays also revealed no interaction between RPSA and the PEDV spike protein (Fig. 2J). Taken together, these results suggest that although RPSA is a critical host factor for PEDV infection, it does not function as a receptor for the virus.

### RPSA KO inhibits the replication stage of PEDV

**Fig 3:**
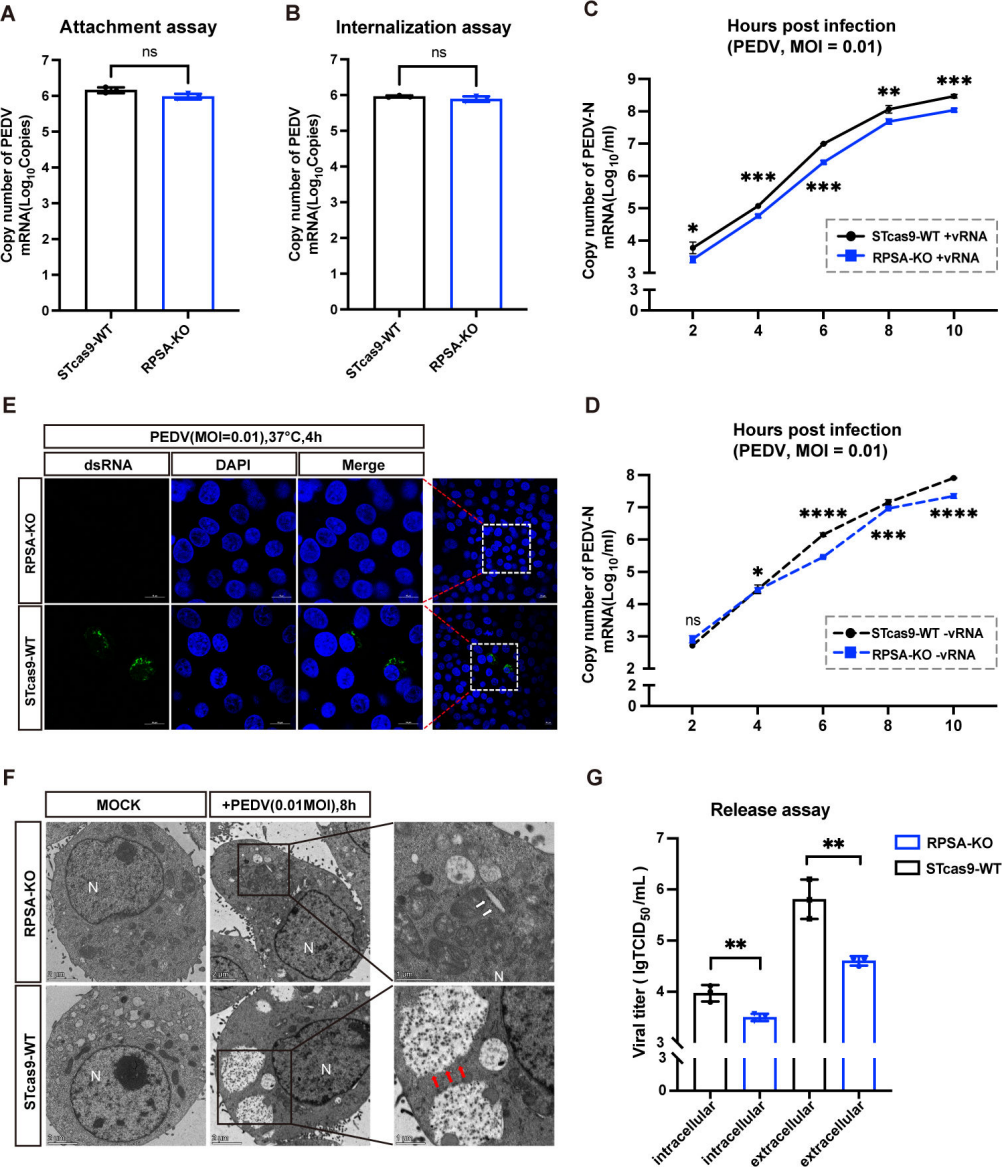
RPSA KO inhibits the replication stage of PEDV. (**A**) RPSA-KO and WT cells were infected with PEDV-YN144 (1.25 × 10^7 particles) at 4°C for 1 h and assessed for PEDV adsorption. The cells were harvested, and viral RNA was extracted to determine virion attachment at the cell surface. (**B**) RPSA-KO cells and WT cells were infected with PEDV-YN144 (1.25 × 10^7 particles) at 4°C for 1 h, followed by incubation at 37°C and acid washing. The ability of PEDV to be internalized by RPSA-KO and WT cells was evaluated via absolute quantitative real-time PCR. (**C and D**) RPSA-KO and WT cells were infected with PEDV-YN144 at an MOI of 0.01. Positive (+vRNA) and negative (−vRNA) viral RNA were quantified via RT-qPCR. (**E**) Confocal microscopy analysis was used to evaluate the replication stage of PEDV by detecting dsRNA formation in WT and RPSA-KO cells at 4 hpi with PEDV-YN144 (MOI of 0.01). Scale bar, 10 µm. (**F**) The effects of RPSA-KO cells on viral particle assembly were evaluated via TEM at 8 hpi. Unlike the abundant viral particles (red arrows) within WT cells, the virus-like particles (white arrows) in RPSA-KO cells were scarce. N, nuclear; scale bars, 1 µm or 2 µm. (**G**) PEDV release in RPSA-KO and WT cells infected with PEDV-YN144 (MOI of 0.01) was assessed. The intracellular and extracellular viral titers at 24 hpi were assessed via virus TCID_50_ assays. The means and SDs of the results from three independent experiments are shown. ns, not significant; *P < 0.05; **P < 0.01; ***P < 0.001; ****P < 0.0001.

To explore the role of RPSA in the PEDV infection cycle, we first examined whether RPSA affects virus attachment or entry. In the attachment assay, RPSA-KO and WT cells were incubated with PEDV for 1 h at 4°C to ensure that the virus had attached to the cell surface. After 15 min of virus absorption at 37°C, we used acidic PBS to remove the virus particles present on the cell surface in the internalization assay. The attachment and adsorption efficiency were detected via RT-qPCR. This showed that the deletion of RPSA did not affect the attachment or adsorption ability of the virus particles, which is consistent with the previous conclusion that RPSA does not play a receptor role in PEDV infection (Fig. 3A and B).

To determine whether RPSA regulates replication of the PEDV genome, we performed strand-specific RT-qPCR to distinguish the production of positive-strand and negative-strand viral RNA (+vRNA and −vRNA, respectively). The production of intracellular +vRNA was remarkably inhibited in RPSA-KO cells compared to WT cells after 4 hpi, while the inhibitory effect of intracellular −vRNA occurred at 6 hpi (Fig. 3C and D). Confocal microscopy revealed that the synthesis of double-stranded RNA (dsRNA), a marker of the PEDV RNA genome, was inhibited in RPAS-KO cells at 4 h post-PEDV infection (Fig. 3E). We then evaluated the effect of RPSA KO on virus particle formation by transmission electron microscopy (TEM). The results showed that viral particle formation was rare in RPSA-KO cells, as indicated by the white arrow, suggesting that RPSA KO severely impaired virion production (Fig. 3F). As expected, the viral release assay showed that both extracellular and intracellular viral titers of RPSA-KO cells were lower than those of WT cells (Fig. 3G). Taken together, these results suggest that RPSA KO suppresses the replication stage of PEDV.

### Silencing RPSA inhibits the ERK1/2 signaling pathway and reduces PEDV replication

**Fig 4:**
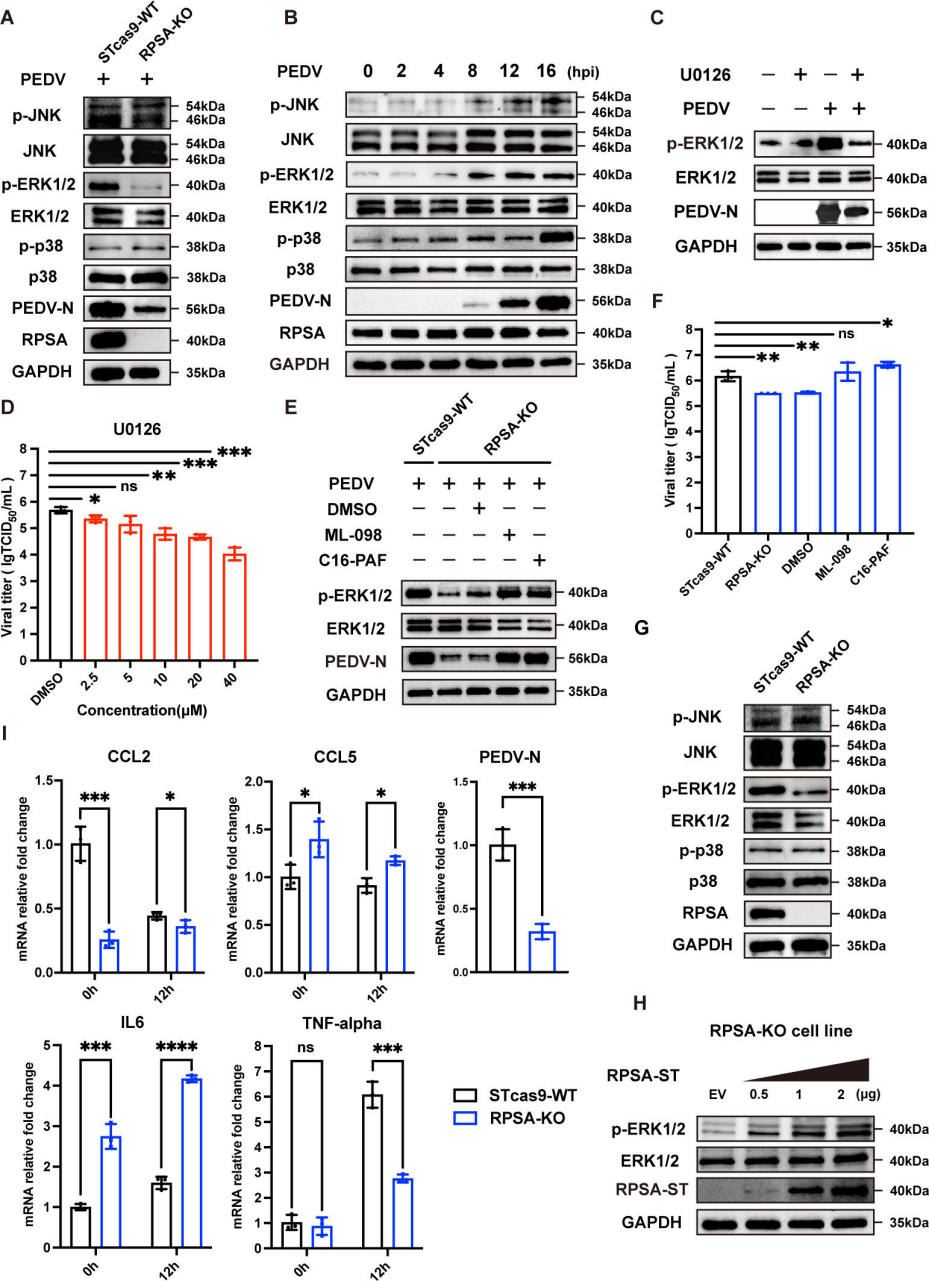
RPSA KO inhibits ERK1/2 signaling to reduce PEDV infection. (**A**) The phosphorylation and total protein expression of JNK, ERK1/2, and p38 in WT and RPSA-KO cells following PEDV-YN144 infection (MOI of 0.01) at 12 hpi were detected by western blotting. (**B**) Temporal activation of three MAPK pathways in PEDV-infected ST cells. ST cells were infected with PEDV-YN144 at an MOI of 0.01 and harvested at 0, 2, 4, 8, 12, and 16 hpi. Representative western blots with the indicated antibodies are shown. (**C**) ST cells were pretreated with DMSO (solvent control) or 40 µM U0126 for 1 h and then infected with PEDV-YN144 for 12 h. The expression of p-ERK1/2, ERK1/2, and PEDV N proteins was detected via western blotting. (**D**) ST cells were preincubated with different concentrations of the ERK1/2 inhibitor U0126 (2.5, 5, 10, 20, 40 µM) for 1 h, followed by PEDV-YN144 infection (MOI of 0.01). The PEDV titers were determined at 12 hpi. (**E and F**) RPSA-KO cells were incubated with 12.5 µM ML-098 or 12.5 µM C16-PAF for 1 h and then infected with PEDV-YN144 at an MOI of 0.01 for 12 h. Western blot assays (**E**) were used to determine the levels of p-ERK1/2, ERK1/2, and PEDV N proteins, and the PEDV titers were measured via TCID_50_ assays (**F**). ST cells and DMSO-treated RPSA-KO cells following PEDV infection served as positive and negative controls, respectively. (**G**) Expression levels of JNK, ERK1/2, p38, p-JNK, p-ERK1/2, and p-p38 in WT and RPSA-KO cells were tested via western blotting. (**H**) RPSA-KO cells were transfected with 2 µg of empty vector or 0.5 µg or 1 µg or 2 µg of pcDNA3.1-RPSA-ST-Tag plasmids for 24 h. Expression levels of ERK1/2 and p-ERK1/2 were tested via western blotting. EV, empty vector. (**I**) RPSA-KO and WT cells were infected with PEDV-YN144 (MOI of 0.01) for 0 or 12 h. RT-qPCR was used to measure the mRNA expression levels of CCL2, CCL5, IL6, TNF-α, and PEDV N protein. The means and SDs of the results from three independent experiments are shown. ns, not significant; *P < 0.05; **P < 0.01; ***P < 0.001; ****P < 0.0001.

Previous studies have shown that MAPK pathways are essential for PEDV replication and that RPSA can modulate MAPK signaling pathway (18). However, it remains unclear whether RPSA knockout inhibits PEDV replication by regulating the MAPK signaling. To elucidate the mechanism underlying the pro-PEDV effect of RPSA, we first assessed the phosphorylation levels of ERK1/2, JNK, and p38 kinases in PEDV-infected cells via Western blotting. Consistent with the level of the PEDV N protein, the phosphorylation of ERK1/2, but not that of JNK or p38, was significantly reduced in RPSA-KO cells compared to WT cells (Fig. 4A). We next examined the MAPK signaling status in STcas9 cells during PEDV infection. As shown in Fig. 4B, the phosphorylation of ERK1/2 started at 4 hpi, while that of JNK and p38 began at 8 hpi, indicating their activation in response to PEDV infection. To further investigate the role of ERK1/2 signaling in PEDV infection, STcas9 cells were pretreated with U0126, a specific inhibitor of ERK1/2 activation (31). Cell proliferation was not affected by 40 µM U0126 (Fig. S2A), but PEDV replication was significantly reduced, as evidenced by Western blotting and TCID_50_ assays (Fig. 4C and D). These results suggest that ERK1/2 activation is crucial for PEDV infection in ST cells.

Furthermore, we evaluated whether the inhibition of PEDV replication in RPSA-KO cells was due to the suppression of p-ERK1/2. RPSA-KO cells were pretreated with the ERK1/2 activator ML-098 (Ras activator [32]) or C16-PAF (ERK1/2 activator [33]) and then infected with PEDV. Compared with WT cells or DMSO-treated RPSA-KO cells, pretreatment with ML-098 or C16-PAF significantly restored PEDV replication in RPSA-KO cells, as indicated by Western blotting and TCID_50_ assays (Fig. 4E and F). We also examined the levels of total and phosphorylated ERK1/2, JNK, and p38 in RPSA-KO and WT cells in the absence of PEDV infection. As expected, RPSA knockout specifically decreased p-ERK1/2 levels (Fig. 4G). We also observed a significant rescue of p-ERK1/2 in RPSA-overexpressing cells compared to empty-vector-transfected cells in a dose-dependent manner (Fig. 4H). These findings suggest that the effect of RPSA on PEDV replication depends on its positive regulation of ERK1/2 signaling.

Since activation of the MAPK signaling pathway by virus infection leads to the expression of proinflammatory cytokines and chemokines (34), we next evaluated the expression of these factors in PEDV-infected WT and RPSA-KO cells. Total RNA was extracted from infected cells, and the transcript levels of CCL2, CCL5, interleukin-6 (IL-6), tumor necrosis factor alpha (TNF-α), and PEDV-N protein were measured by RT-qPCR. The results showed that the knockout of RPSA impaired the expression of CCL2 and TNF-α, which was accompanied by a decrease in PEDV-N protein levels (Fig. 4I). Unexpectedly, the expression of CCL5 and IL-6 was increased in RPSA-KO cells. We also evaluated cytokine levels in PEDV-infected cells pretreated with U0126. With the exception of a decrease in IL-6, all other proinflammatory factors showed a similar trend as in RPSA-KO cells (Fig. S2D). These results suggest that RPSA affects the expression of inflammatory cytokines during PEDV infection via the ERK1/2 signaling pathway.

### RPSA is involved in the process of lipid synthesis and transport during PEDV infection

**Fig 5:**
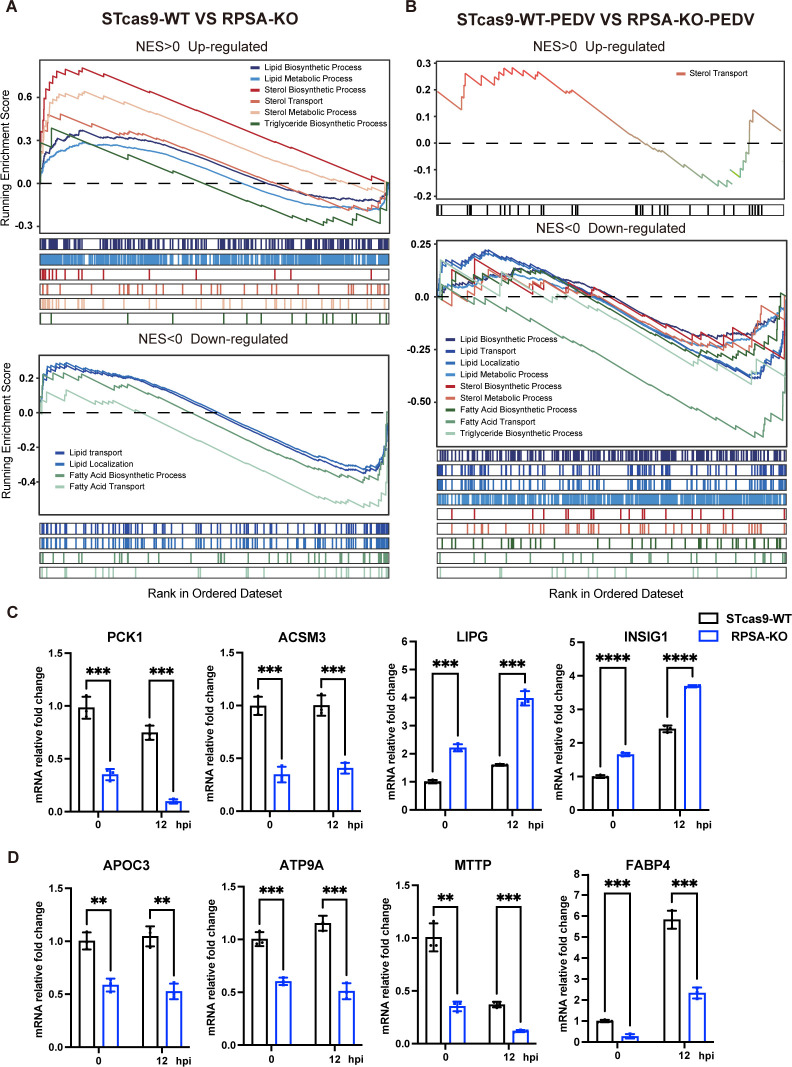
RPSA is a positive regulator of lipid biosynthesis and transport during PEDV infection. (**A and B**) Gene Set Enrichment Analysis (GSEA) of differentially expressed genes from the RNA-seq data showing a distinct downregulation of multiple lipid-metabolism-related biological processes in two pairwise groups: (**A**) WT-MOCK vs RPSA-KO-MOCK; (**B**) WT-PEDV vs RPSA-KO-PEDV. Enhanced or diminished biological processes are distinguished on the basis of the Normalized Enrichment Score (NES). (**C**) RT-qPCR validation of the mRNA expression of lipid-biosynthesis factors, including *PCK1*, *ACSM3*, *LIPG,* and *INSIG1*, according to the RNA-seq results. (**D**) RT-qPCR analysis showing a significant reduction in the expression of lipid transport-associated genes following RPSA deletion in ST cells. The data are representative of at least three independent experiments. *P* values were determined by two-sided Student’s *t*-test. **P < 0.01; ***P < 0.001; ****P < 0.0001.

To comprehensively explore the biological effects mediated by RPSA knockout and their potential role in inhibiting PEDV replication, we performed RNA sequencing (RNA-seq) using total RNA extracted from uninfected and PEDV-infected RPSA-KO and WT cells. The reliability between duplicate samples was assessed, and the selected sample was considered representative (Fig. S3A). Interestingly, we observed a notable decrease in the mRNA transcript levels of APN in RPSA-KO cells (Fig. S4A and B). Additionally, Gene Ontology (GO) and Kyoto Encyclopedia of Genes and Genomes (KEGG) analyses of differentially expressed genes revealed that lipid metabolism was the most enriched biological process under both uninfected and PEDV-infected conditions (Fig. S5 to S8). Furthermore, we focused on 10 biological processes related to lipid metabolism in Gene Set Enrichment Analysis (GSEA). These results indicated that the suppression of lipid localization and lipid transport was correlated with the decrease of RPSA. Moreover, eight other metabolic processes, including sterol biosynthesis and triglyceride biosynthesis, which are important for PEDV replication, were reduced in PEDV-infected RPSA-KO cells, except for sterol transport (Fig. 5A and B).

To confirm these results, we selected genes involved in lipid synthesis, such as PCK1, ACSM3, LIPG, and INSIG1, and performed quantitative reverse transcription PCR (RT-qPCR). Consistent with the RNA-seq data, we observed a decrease in *PCK1* and *ACSM3*, which are positive regulators of lipid synthesis, and an increase in *LIPG* and *INSIG1*, which are involved in lipid decomposition (Fig. 5C). Additionally, we assessed the expression of genes responsible for lipid transport, including *APOC3*, *ATP9A*, *MTTP,* and *FABP4*. These genes were significantly downregulated, further confirming the suppression of lipid transport observed in the RNA-seq data (Fig. 5D). Overall, these results suggest that RPSA plays a critical role in lipid metabolic processes induced by PEDV infection according to the transcriptome analysis.

### RPSA knockout partially relies on ERK1/2 signaling to reduce lipid accumulation induced by PEDV replication

**Fig 6:**
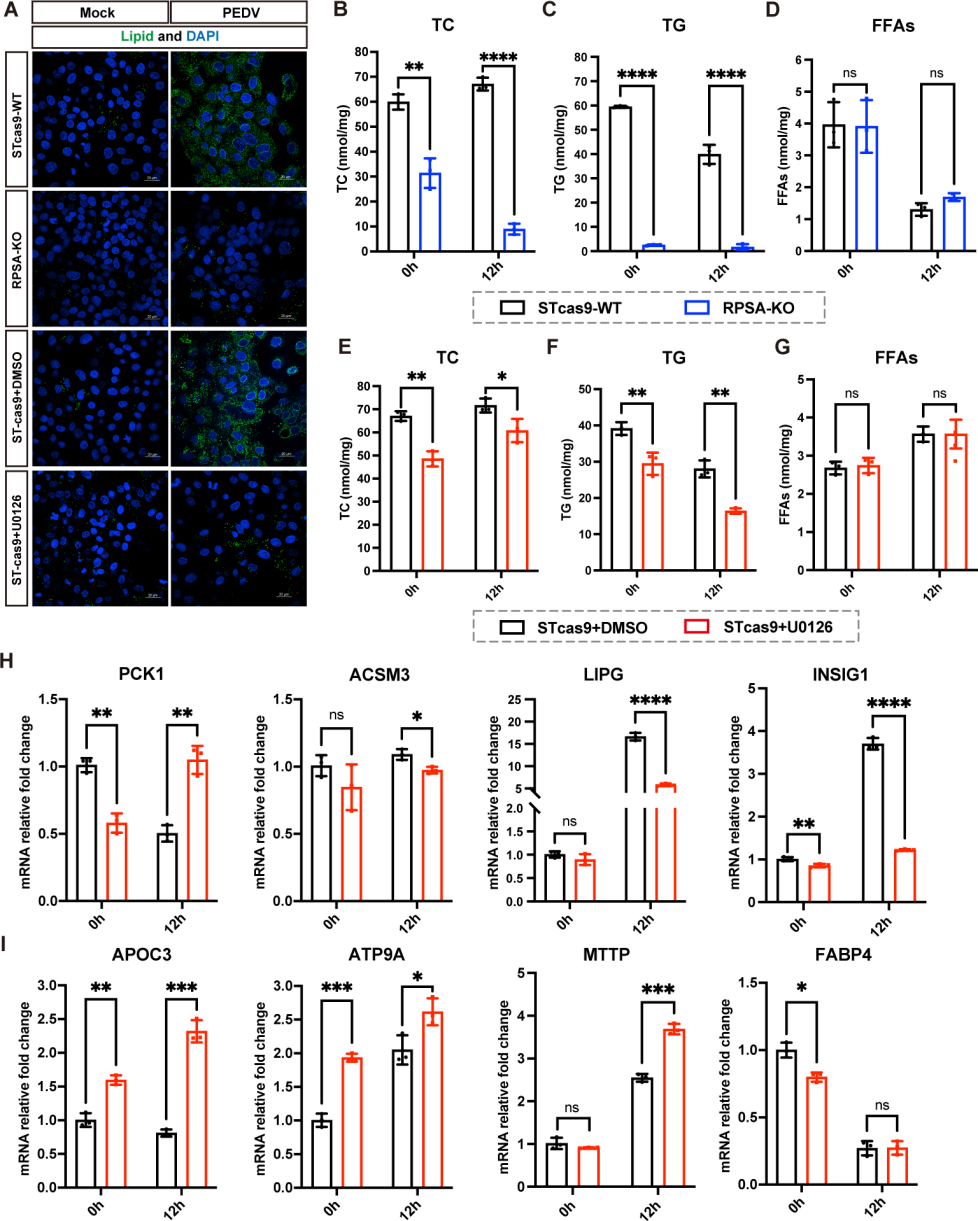
RPSA partially relies on ERK1/2 signaling to impair the lipid accumulation to inhibit viral replication. (**A**) Confocal microscopy analysis of LDs formation was performed to assess the lipid accumulation in RPSA-KO, WT, DSMO-treated, and U0126-treated cells following infection with PEDV-YN144 (MOI = 0.01) at 12 hpi. Scale bar, 20 µm. (**B−D**) TC (**B**), TG (**C**), and FFAs (**D**) were quantified in RPSA-KO and WT cells via biochemical kits following infection with PEDV-YN144 at an MOI of 0.01 at 0 and 12 h. (**E−G**) Quantification of TC (**E**), TG (**F**), and FFAs (**G**) levels in DMSO-treated and U0126-treated cells infected with PEDV-YN144 at 0.01 MOI at 0 and 12 hpi, determined using biochemical kits. (**H and I**) ST cells were pretreated with DMSO (solvent control) or 40 µM U0126 for 1 h and then infected with PEDV-YN144 for 0 and 12 h. The mRNA expression levels of host factors involved in lipid biosynthesis (**H**) and lipid transport (**I**) were quantified by RT-qPCR assay. The means and SDs of the results from three independent experiments are shown. ns, not significant; *P < 0.05; **P < 0.01; ***P < 0.001; ****P < 0.0001.

Lipid droplets (LDs) are organelles that store neutral lipids, such as triglycerides and sterol esters, and their accumulation can facilitate viral proliferation (35). To assess the effect of RPSA knockout on the accumulation of LDs, mock-infected and PEDV-infected RPSA-KO and WT cells were analyzed by confocal microscopy. Compared to WT cells, a significant reduction in the accumulation of cellular LDs was observed in RPSA-KO cells during PEDV infection (Fig. 6A). We further quantified the impact on intracellular lipid profiles and found declines in total cholesterol (TC) and triglyceride (TG) levels in RPSA-KO cells following PEDV infection (Fig. 6B and C). No differences were observed in the synthesis of free fatty acids (FFAs) between RPSA-KO and WT cells (Fig. 6D). These results indicate that RPSA knockout in ST cells impairs PEDV-induced lipid synthesis and accumulation.

To investigate whether the effect of RPSA knockout on lipid biosynthesis depends on the ERK1/2 pathway, we examined the formation of LDs and lipid profiles in mock-infected and PEDV-infected cells treated with DMSO or U0126, an ERK1/2 inhibitor. Consistent with the effects of RPSA deletion, the formation of LDs and the synthesis of TC and TG were significantly reduced in U0126-treated cells compared with WT cells at 12 hpi, suggesting that inhibiting the ERK1/2 pathway also affects lipid synthesis and accumulation induced by PEDV replication (Fig. 6A, E thorugh G). We next examined whether the effects of RPSA deletion and ERK1/2 inhibition on lipid metabolism are mediated by the same factors associated with lipid synthesis. We quantified the levels of *PCK1*, *ACSM3*, *LIPG,* and *INSIG1* in DMSO- and U0126-treated cells at 0 and 12 hpi by RT-qPCR. In contrast to the factors tested in infected RPSA-KO cells, treatment with U0126 resulted in higher mRNA expression of *PCK1* and lower levels of *LIPG* and *INSIG1* (Fig. 6H). We also assessed the impact of decreased p-ERK1/2 on lipid-transport-related factors, including *APOC3*, *ATP9A*, *MTTP,* and *FABP4*. Inhibition of the ERK1/2 signaling pathway led to an increase in the expression of *APOC3*, *ATP9A,* and *MTTP* (Fig. 6I). The contrasting trends in factors related to lipid synthesis and transport between infected RPSA-KO cells and U0126-treated cells suggest that RPSA deletion and ERK1/2 inhibition impair lipid metabolism during PEDV infection via different mechanisms. Taken together, the deletion of RPSA appears to regulate intracellular lipid accumulation in a complicated manner to impair PEDV replication, one of the mechanisms being inhibition of the ERK1/2 signaling pathway.

### RPSA is a host factor for the replication of several porcine enteric coronaviruses

**Fig 7:**
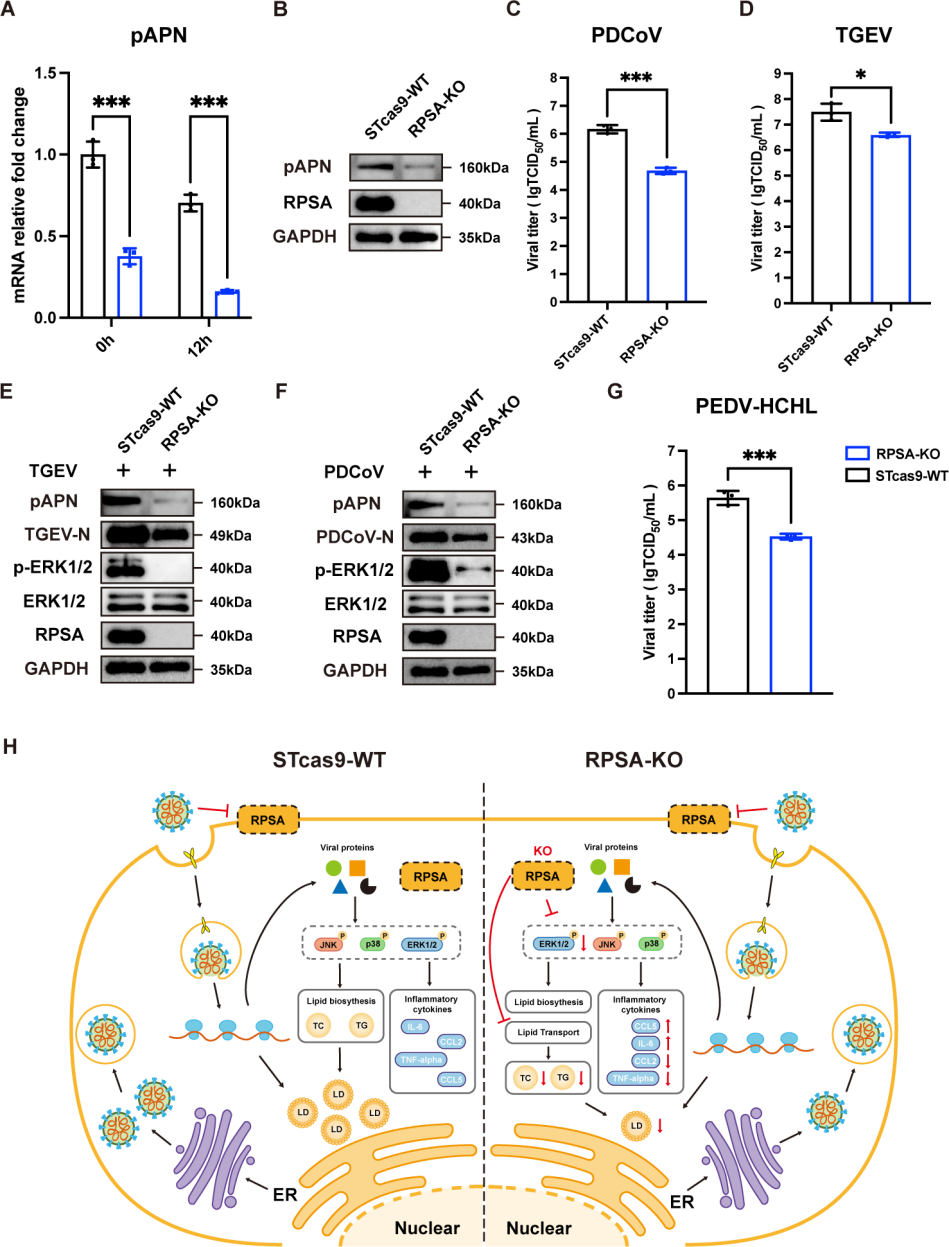
RPSA is a host factor for multiple porcine enteric coronaviruses. (**A**) RT-qPCR analysis for determination of relative mRNA levels of APN in RPSA-KO and WT cells following PEDV-YN144 infection at 0.01 MOI at 0 and 12 hpi. (**B**) Western blot analysis of APN expression level in RPSA-KO and WT cells. (**C and D**) TCID_50_ assay to measure viral titers in WT and RPSA KO ST cells infected with (**C**) PDCoV (MO of 0.01, 12 hpi) and (**D**) TGEV (MOI of 0.01, 18 hpi). (**E and F**) Western blot analysis of APN, ERK1/2, p-ERK1/2, and viral N proteins in WT and RPSA KO cells infected with PDCoV (MOI = 0.01, 12 hpi) (**F**) or TGEV (MOI = 0.01, 18 hpi) (**E**). (**G**) PEDV titers in WT and RPSA KO ST cells infected with the PEDV-HCHL strain (G2a subtype, MOI = 0.01, 12 hpi) were determined using the TCID_50_ assay. (**H**) Proposed model illustrating the role of RPSA in the CoV replication cycle is shown. PEDV does not utilize RPSA located on the cell membrane surface as a receptor. In WT cells, CoVs hijack lipid biogenesis and regulate inflammatory response, partly through the ERK1/2 pathway, to facilitate replication. In RPSA-KO cells, the inhibition of ERK1/2 signaling affected the pro-inflammatory cytokines and chemokines, while decreased p-ERK1/2 levels impair the biosynthesis of total cholesterol and triglycerides, leading to reduced lipid droplet (LD) formation. Additionally, the impaired lipid transport and reduced LD accumulation in RPSA KO cells hinder viral replication.

As mentioned above, knockout of RPSA significantly downregulated the expression of APN according to the RNA-seq data. To further validate this finding, we assessed APN expression in uninfected and PEDV-infected RPSA-KO and WT cells by RT-qPCR and Western blotting. APN expression was significantly reduced at both the mRNA and protein levels, which correlated with a decrease in RPSA (Fig. 7A and B). Given that APN is a known functional receptor involved in the replication of TGEV and PDCoV (36, 37), two porcine enteric coronaviruses, we examined the possible effects of RPSA on these viruses. Compared to WT cells, viral titers of TGEV and PDCoV were significantly decreased in RPSA-KO cells (Fig. 7C and D). We also investigated the status of ERK1/2 signaling in RPSA-KO and WT cells during TGEV and PDCoV infection. Consistent with the decreased expression of the N protein of the corresponding coronaviruses, p-ERK1/2 levels were lower in RPSA-KO cells during both TGEV and PDCoV replication, suggesting that the ERK1/2 pathway was inhibited (Fig. 7E and F). Additionally, we confirmed that RPSA KO significantly reduced the replication ability of the PEDV-HCHL strain belonging to the GII-a subtype, as measured by the virus titration assay (Fig. 7G). Overall, these results suggest that RPSA is an essential host factor that limits the infection of porcine enteric coronaviruses by either inhibiting the ERK1/2 signaling pathway or reducing APN expression.

## DISCUSSION

Emerging coronaviruses (CoVs) pose a significant threat to both human and animal health worldwide. This emphasizes the urgent need for further research into the interaction between CoVs and host factors to identify novel drug targets for developing therapeutics against these viruses. Genome-wide CRISPR/Cas9 screens of Vero E6 or HEK-293T cells have identified several key host factors, such as TRIM2, SLC35A1, and PKCθ, associated with PEDV infection (38, 39). However, species and cellular differences between non-porcine-derived and porcine-derived cells limited the screening results. Since PEDV has no known receptor, genome-wide screening might hinder the search for its receptor. In this study, we first constructed a sub-genome-wide CRISPR/Cas9 library specifically targeting cellular membrane proteins in LLC-PK1 cells to screen for essential membrane proteins, including potential receptors, involved in PEDV infection. Experimental validation of hits from our CRISPR screen revealed RPSA and ROBO4 as novel host proteins involved in the PEDV life cycle. Other candidate genes will be explored in the subsequent research conducted in our laboratory. Given the role of RPSA as a receptor in various viral infections, we focused on it for further research and systematically studied the possible conservative mechanism of RPSA in porcine enteric coronavirus replication.

RPSA, also known as laminin receptor 1, is ubiquitously expressed and plays multiple roles in viral infections. Previous studies have identified RPSA as a receptor for several viruses (27–29, 40). For example, dengue virus serotype 1 binds to RPSA expressed on liver cells to facilitate viral entry (28), and screening of small interfering RNAs (siRNAs) against membrane proteins revealed that RPSA interacts with the Erns protein of classical swine fever virus (29). However, in our study, incubation of cells with an antibody targeting the extracellular C-terminus of RPSA did not block PEDV infection, and there was no strong interaction between RPSA and the PEDV spike (S) protein. Furthermore, overexpression of RPSA did not render PK15 cells susceptible to PEDV infection. These results suggest that RPSA is not a cellular receptor for PEDV entry.

Our findings also demonstrated that RPSA regulates PEDV replication through the ERK1/2 pathway rather than through the JNK or p38 signaling pathways. Coronaviruses, including SARS-CoV-2, MERS-CoV, and HCoV-229E, preferentially manipulate MAPK pathways to promote their replication (41–44). Consistent with these findings, our study revealed that ERK1/2 signaling is essential for the efficient replication of PEDV, TGEV, and PDCoV in ST cells, indicating that MAPKs play a conserved role in CoV replication (21, 22, 45, 46). The role of RPSA in the regulation of MAPK pathways is complex. On the one hand, RPSA negatively regulates the activation of three MAPK cascades during FMDV infection, exhibiting antiviral activity (20). On the other hand, inhibition of RPSA leads to a reduction of p-ERK1/2 levels in pancreatic cancer cells (47). In our study, RPSA induced the phosphorylation of ERK1/2 in a dose-dependent manner, consistent with positive regulation in tumor cell lines. We hypothesize that the abundance, species, and subcellular localization of RPSA may contribute to its divergent regulation of MAPK signaling. Given that exogenous laminin, a ligand of RPSA, activates the ERK1/2 cascade (48). It is possible that RPSA functions as a receptor for laminin and that the absence of RPSA may disrupt laminin-induced signals, which impairs ERK1/2 activation and, thus, inhibits PEDV infection. However, the exact mechanisms remain to be investigated.

Among the genes differentially expressed during PEDV infection, host factors related to lipid metabolism were significantly altered in RPSA-knockout cells. Previous studies have shown that coronavirus infection triggers reprogramming of host lipid metabolism to support palmitoylation of viral proteins and formation of double-membrane vesicles, which are crucial for virus replication (49–52). Additionally, genes involved in cholesterol regulation, such as SREBP2 and FXR, are upregulated during PEDV infection (53). We observed an increase in lipid droplets (LDs) during PEDV infection, potentially supporting viral replication. RNA sequencing revealed that RPSA affects a number of host factors involved in lipid biosynthesis and transport, which has not been previously reported. Moreover, RPSA knockout suppressed PEDV replication by reducing the cellular lipid content, raising questions about the mechanisms by which RPSA regulates lipid metabolism. Previous studies have suggested that the regulatory effect of ERK1/2 signaling on lipid metabolism may depend on the lipid droplet composition in various cells (54–56). Our inhibitor assay showed that U0126 reduced the lipid content and downregulated the mRNA levels of genes involved in lipid biogenesis but not lipid transport. Due to the complexity of RPSA’s role, the exact mechanisms by which it modulates lipid transport require further investigation. Given the high conservation of RPSA (57), our discovery provides a new target for further research on how lipid metabolism can be influenced to limit viral infection.

Interestingly, we found that RPSA positively regulates the expression of APN and, thus, affects TGEV and PDCoV infection in ST cells. APN has been identified as a functional receptor for several CoVs, including HCoV-229E, FCoV, TGEV, and PDCoV (58, 59). It is, therefore, plausible that RPSA acts as a conserved host factor in the replication of these viruses. Notably, the inhibition of APN has been reported to enhance ERK1/2 signaling in various cancer models (60, 61), suggesting that RPSA may regulate the ERK1/2 pathway independently of APN. However, the molecular mechanisms underlying RPSA-mediated regulation of APN remain unclear and warrant further investigation.

In summary, our study has identified RPSA as a novel host factor that regulates PEDV replication. Although RPSA is not a receptor for PEDV entry, it plays a crucial role in regulating ERK1/2 signaling, lipid metabolism, and APN expression. As shown in Fig. 7H, RPSA knockout inhibited ERK1/2 signaling, interfered with lipid synthesis and transport, and significantly reduced APN expression. Collectively, these results suggest that RPSA has broad antiviral effects against porcine enteric coronaviruses and may serve as a host-directed target to inhibit the replication of emerging and re-emerging CoVs.

## MATERIALS AND METHODS

### Plasmids

Lentiviral sgRNA expression vectors were constructed by digesting the sgLenti vector with *AarI* enzyme (Thermo Fisher Scientific, USA). Paired oligonucleotides corresponding to the sgRNAs were annealed and cloned and inserted into the linearized vector. For rescue and overexpression experiments, the coding sequence of RPSA gene (Gene ID: 641351) was amplified from the porcine genome cDNA, and synonymous codon mutations were introduced into the sgRNA target sequence in RPSA-KO cells to exclude the effects of the sgRNA and Cas9. The mutated sequence was subsequently cloned and inserted into the pQCXIP-Flag and pcDNA3.1-ST vector via MultiF Seamless Assembly (ABclonal, RK21020, China). The plasmids were confirmed by Sanger sequencing (Tsingke). The primer sequences are listed in Table 1.

**TABLE 1:** Primer pairs and sgRNAs for plasmids construction

Primer	Sequence
RPSA-sgRNA-F	TTGGCGCCAACAGAAGTTTCTCCC
RPSA-sgRNA-R	AAACGGGAGAAACTTCTGTTGGCG
RPSA-CDS-F	ATGTCCGGAGCCCTCGATGT
RPSA-CDS-R	AGACCACTCAGTGGTTGTTC
RPSA-TB-F	GGGAGAAGCTACTATTGGCT
RPSA-TB-R	AGCCAATAGTAGCTTCTCCC

### Cell culture and viruses

Swine testicular (ST), human embryonic kidney 293T (HEK-293T), pig kidney (LLC-PK1 and PK-15), and African green monkey kidney (Vero-CCL81) cells were purchased from the Cell Bank of the Chinese Academy of Sciences (Shanghai, China). Cas9-expressing cell lines ST-cas9 and PK1-cas9 were generated via lenti-Cas9-Blast transduction and were preserved in our laboratory. All cells were cultured in Dulbecco’s modified Eagle medium (DMEM; Gibco, USA) supplemented with 10% fetal bovine serum (Excell Bio, Shanghai, China), 100 U/mL penicillin, and 100 µg/mL streptomycin (Gibco, USA) at 37°C with 5% CO_2_. All cell lines were confirmed to be mycoplasma-free. The following viruses were used: the TGEV WH-1 strain (GenBank accession no. HQ462571.1), the PEDV-YN144 strain (GenBank accession no. KT021232.1), and the PEDV-HCHL strain (GenBank accession no. OR722805.1). PDCoV-GFP and PEDV-GDU-GFP were generated using a reverse genetics system as described previously (62–64). PEDV was propagated in Vero-CCL81 cells, and PDCoV and TGEV were propagated in LLC-PK1 cells.

### Porcine membrane-protein-scale sgRNA library design

Proteins with subcellular localization to the cell membrane were obtained from the Uniprot website (https://www.uniprot.org). Sequences of protein-coding genes were acquired from the Ensembl database (http://www.ensembl.org/index.html). Seven sgRNAs were designed against each membrane-protein-coding gene using CRISPR-offinder software (http://www.biootools.com). Briefly, the selected sgRNAs were weighted based on targeting the first 50% of the open reading frames and minimizing potential off-target sites. The maximum number of mismatches allowed up to three nucleotides to the DNA target in the 20-mer target region of the selected sgRNAs, and sgRNAs with fewer mismatches were preferentially selected. The designed sgRNA library was synthesized by GenScript Biotech Corp. (USA) and then ligated into the linearized lenti-sgRNA-EGFP vector using Gibson Assembly (NEB).

### Membrane protein CRISPR/Cas9 library screen

The membrane-protein-targeted sgRNA library consisting of ±11,000 unique sgRNA sequences was introduced into LLC-PK1-Cas9 cells as previously described (65). The titer of the lentivirus was determined to ensure it reached a 500-fold concentration relative to the library. Transduced cells were selected with 6 µg/mL puromycin (Sigma-Aldrich, USA) to ensure complete selection of sgRNA-transduced cells. Cell counting was subsequently performed to validate that the final library coverage exceeded 500-fold. Cells were subsequently infected with the PEDV-GDU-GFP strain at an MOI of 1 for 3 days, as the first round of virus challenge screening, and cells were washed with DMEM to remove dead cells. The surviving resistant cells were expanded for the deep sequencing analysis as described previously (66) and the next round of infection.

### Construction of candidate gene-KO, stable gene rescue, and overexpression cell lines

Lentiviral sgRNAs targeting each candidate genes were transduced into ST-cas9 or PK1-cas9 cells. After 48 h, the transduced cells were selected with hygromycin (Sigma-Aldrich, USA) for 2 days (6 µg/mL for LLC-PK1-cas9 cells and 8 µg/mL for ST-cas9 cells). To generate rescue and overexpression cell lines, RPSA-KO, ST-cas9, and PK-15 cells were transduced with lentivirus carrying the full-length RPSA gene containing synonymous mutations. After 48 h, the cells were selected with puromycin (Sigma-Aldrich, USA) for 2 days (3 µg/mL for ST-cas9 cells, 4 µg/mL for PK-15 cells) to enrich the lentivirus-transduced cells. Expression of the restored Flag-Tagged RPSA gene was confirmed by western blotting.

### Western blotting and antibodies

Cells were lysed in cell lysis buffer containing an additional 10× phosphatase inhibitor (Biosharp) for 30 min at 4°C, and cell debris was removed via centrifugation (12,000 × *g* for 10 min at 4°C). The lysates were denatured in SDS at 95°C for 10 min, followed by incubation on ice for 5 min. Proteins were separated by SDS-PAGE and transferred onto PVDF membranes. The PVDF membranes were blocked for 2 h with Tris-buffered saline plus Tween-20 (TBST) containing 5% nonfat milk. For the detection of phosphorylated proteins, membranes were instead blocked in 5% BSA buffer for 12 h. Membranes were then incubated with primary antibodies overnight at 4°C or for 2 h at room temperature. The following primary antibodies were used: Flag-tag mouse mAb (AE005, ABclonal), phospho-SPAK/JNK (Thr183/Tyr185) mouse mAb (9255, Cell Signaling Technology), JNK recombinant antibody (81629-1-RR, Proteintech), phospho-p38 (Thr180/Tyr182) rabbit pAb (29796-1-AP, Proteintech), p38 MAPK rabbit pAb (14064-1-AP, Proteintech), phospho-ERK1/2 (Thr202/Tyr204) rabbit mAb (110441-R0072, SinoBiological), ERK1/2 rabbit pAb (11257-1-AP, Proteintech), GAPDH mouse mAb (AC002, ABclonal), and RPSA mouse mAb (67324-1-Ig, Proteintech). PEDV-N mouse antibody and Strep-II tag (ST-Tag) mouse antibody were generated and preserved in our laboratory. The secondary antibodies were used at a 1:5,000 dilution for 1 h at room temperature. Proteins were detected using Western ECL substrate detection reagents (Bio-Rad).

### RT-qPCR

Total RNA was extracted from cells with TRIzol reagent (TransGen Biotech, China). Complementary DNA (cDNA) was synthesized using the HiScript II 1st Strand cDNA Synthesis Kit (R211-01, Vazyme) in a total volume of 20 µL. In strand-specific RT-qPCR assay, strand-specific reverse transcription was performed using strand-specific RT primers for +vRNA and −vRNA, respectively. Each RT-qPCR contained 10 ng of cDNA and 5 µM primer pairs using SYBR Green Mix (RK21203, ABclonal). Amplifications were performed on a CFX96 Real-Time PCR Detection System (Bio-Rad, USA) programmed for one cycle of 5 min at 95°C, followed by 41 cycles of 95°C for 5 s and 60°C for 30 s. Relative gene expression was calculated using the 2^−ΔΔCt^ method with GAPDH as the normalization control. For absolute RT-qPCR, 500 ng of viral RNA was used as the template for cDNA synthesis. Absolute RT-qPCR assays were performed using AceQ qPCR Probe Mix (Q112-02, Vazyme) with primers specific for the M gene of PEDV in a final reaction volume of 20 µL. The PEDV M protein-coding cDNA sequence from GenBank (accession number: KT021228.1) was cloned and inserted into the pcDNA3.1 vector and used as an internal reference for the quantification of PEDV copy numbers. All primers used for reverse transcription and quantitative PCR are listed in Table 2.

**TABLE 2:** Primer used for reverse transcription and quantitative PCR

Primer	Sequence
+vRNA	GAAGCACTTTCTCACTATCTGTG
−vRNA	GTCTGCATTCCAGTGCTTGG
probe-PEDV-M	FAM-TTCGTCACAGTCGCCAAGG-TAMRA
qPCR-PEDV-M-F	CGTACAGGTAAGTCAATTAC
qPCR-PEDV-M-R	GATGAAGCATTGACTGAA
PEDV-N-F	TAACCAGGGTCGTGGAGCT
PEDV-N-R	CACCAGATCATCGCGTGATGT
CCL2-F	TCCAGCATGAAGGTCTCTGC
CCL2-R	ACTGGAGAATTAATTGCATCTGGC
CCL5-F	ATATGCCTCGGACACCACAC
CCL5-R	TGTACTCCCGCACCCATTTC
IL-6-F	CCCTGAGGCAAAAGGGAAAGA
IL-6-R	TGGACGGCATCAATCTCAGG
TNF-alpha-F	TGGCCCAAGGACTCAGATCAT
TNF-alpha-R	ATTGGCATACCCACTCTGCC
PCK1-F	AGGAGAGAAAACGTAGGCGAC
PCK1-R	TGGTTGAGGCCGTTTGAGAG
INSIG1-F	ATCAACCACGCCAGTGCTAA
INSIG1-R	TCCGGGGACGTGTACTGATA
LIPG-F	ACACAGCGTCGCAAGGAT
LIPG-R	CAGGATCCAGACCCGTGATT
ACSM3-F	TACCAGTGGAACAAGCGGAC
ACSM3-R	AAATCCAGCCAGAACCTTCCA
APOC3-F	ACTATGTGAAGCAGGCCACC
APOC3-R	GTTACCCAGCCCCTGGC
ATP9A-F	AAGAAGCGGATGGACAGCAG
ATP9A-R	TGGTTAAACAGCACCCCAGG
MTTP-F	AGCAAAATGGTCCGTCGAGT
MTTP-R	CGAATGGGGACCACGTTCTA
FABP4-F	TGAAAGAAGTGGGAGTGGGC
FABP4-R	CTGGCCCAATTTGAAGGCAA
APN-F	CCTCAGCGTTCGACTACCTC
APN-R	TCAGCAAGACCCAATCGTCC

### Indirect immunofluorescence assay

The expression of PEDV N protein in candidate gene KO and control cells was determined via indirect immunofluorescence assay. Cells were infected with PEDV-YN144 at different MOIs, then fixed, permeabilized, and blocked before incubation with primary antibodies against PEDV N, RPSA, or anti-dsRNA (the human anti-dsRNA antibody was prepared in our laboratory). The secondary antibodies included Alexa Fluor 488 Donkey anti-mouse IgG (H + L) (Antgene, ANT-023, 1:1,000) and Alexa Flur 488 AffiniPure Donkey anti-human IgG (H + L) (Jackson ImmunoResearch, no. 709-545-149, 1:1,000). The cells were incubated with secondary antibodies at 37°C for 1 h. The cell nuclei were stained with 4′,6-diamidino-2-phenylindole (DAPI) (Sigma, USA). Imaging was performed via a fluorescence microscope (Thermo Fisher Scientific EVOS FL Auto).

### Co-Immunoprecipitation assay

To test whether RPSA interacts with the PEDV S protein, HEK293T cells (1 × 10^7^) were seeded in 10 cm cell culture dishes and transfected with pcDNA3.1-RPSA-ST for 24 h. The cells were lysed using lysis buffer (Beyotime, China) at 4°C for 30 min. Subsequently, the lysates were centrifuged at 12,000 × *g* for 10 min. The supernatant was then incubated with protein A/G-conjugated agarose beads at 4°C for 1 h to minimize non-specific binding. PEDV-S-pFc protein and IgG were incubated with protein A/G-conjugated agarose beads at 4°C for 4 h to precipitate the Fc-tagged PEDV S protein or IgG. A portion of the supernatant of the lysed cells was kept for the whole-cell extract assay. The remaining supernatant was immunoprecipitated with Fc-tagged PEDV S protein or IgG overnight at 4°C. The beads were washed five times with ice-cold lysis buffer, boiled for 10 min in SDS-PAGE loading buffer, and then subjected to western blotting.

### Virion attachment and internalization assay

WT and RPSA-KO cells were infected with PEDV-YN144 (1.25 × 10^7 particles) at 4°C for 1 h. For the attachment assay, cells were washed three times with cold PBS to remove unbound viral particles and harvested for viral RNA extraction. The amount of viral RNA was quantified by RT-qPCR. For the internalization assay, the infected cells described above were further cultured with pre-warmed DMEM at 37°C for 15 min to allow internalization. Subsequently, the cells were washed three times with cold PBS (pH 3.0) to remove uninternalized viral particles. Total cellular RNA was extracted using TRIzol reagent, and the internalized viral RNA was quantified by RT-qPCR.

### Confocal microscopy

To investigate whether the replication stage of PEDV was affected, equal numbers of RPSA-KO and WT cells were seeded in 35 mm Petri dishes (Biosharp, China) and cultured overnight. Cells were infected with PEDV-YN144 (MOI of 0.01) and incubated at 37°C for 4 h to observe the replication stage of PEDV. An indirect immunofluorescence assay was performed as described above, and images were acquired using a laser scanning confocal microscope (Nikon). To assess the formation of LDs, cells were incubated with PEDV-YN144 (MOI of 0.01) for 12 hours. The LDs were stained with BODIPY 493/503 (Thermo Fisher Scientific, USA) for 20 min, and the nuclei were stained with DAPI for 10 min and imaged to determine the number of LDs.

### TEM assay

The cells were infected with PEDV-YN144 at an MOI of 0.01 for 8 h. The cells were washed three times with precooled PBS and fixed with 1.5 mL of 2.5% glutaraldehyde (Servicebio) at room temperature for 2 h. TEM assay was performed by Servicebio Company, and images were taken via an HZAU TEM platform (Thermo Fisher Scientific Talos L120C G2).

### Drug treatment assay

U0126 (cat. no. HY-12031A), ML-098 (cat. no. HY-19800), and C16-PAF (cat. no. HY-108635) were purchased from MedChemExpress, dissolved in dimethyl sulfoxide at a stock concentration of 5 mM, and stored in aliquots at −80°C. Cells were pretreated with the specified concentration of each drug for 2 h before infection with PEDV-YN144 at an MOI of 0.01. The cells were harvested at the indicated time points for subsequent western blotting, viral titer assays, RT-qPCR, and immunofluorescence assays.

### RNA sequencing and transcriptome analysis

For high-throughput RNA sequencing, RNA libraries were prepared from different experimental groups, including RPSA-KO cells (i.e., RPSA-KO-MOCK, RPSA-KO-PEDV) and WT cells (i.e., WT-MOCK, WT-PEDV), with three replicates for each group. Poly (A) + RNA isolation, library construction, and sequencing were performed by an external company. Sequence quality of all the samples was assessed usingFastQC, and quality was trimmed using Trimmomatic. The trimmed reads were aligned to the Sus scrofa reference genome (v11.1) using HISAT2 (v 2.2.1). Gene expression levels were quantified by calculating fragments per kilobase of exon per million mapped reads (FPKM) for each unigene via FeatureCounts (v2.0.8). Differentially expressed genes (DEGs) were identified via DESeq2 (v1.46.0) with a significance threshold of *P*-value ≤ 0.05 and an absolute fold-change ≥2. DEG analysis for different pairwise comparisons was subsequently performed to identify enriched pathways using KEGG and GO analysis with the Cluster Profiler package (v4.14.3).

### Intracellular FFAs, TG, and TC detection assay

Total cellular cholesterol and triglycerides were quantified using the TC assay kit (E1013, Applygen) and the TG assay kit (E1015, Applygen), respectively. RPSA-KO and WT cells in 12-well plates following PEDV-YN144 infection (MOI of 0.01) at 12 hpi were collected and washed three times with PBS. Subsequently, the cells were lysed in 100 µL extraction solution for 10 min at room temperature, followed by heating at 70°C for 5 min. The cellular debris was removed by centrifugation (2,000 × *g* for 5 min at 4°C). Then, 10 µL of the centrifuged sample was transferred into a 96-well plate, and 190 µL of working solution was added, and the plate was shaken well and incubated for 20 min at 37°C while protected from light. Then, the samples were measured at 550 nm using the spectrophotometer (Thermo Fisher Scientific, USA). The cellular free fatty acids were measured with FFAs assay kit (A042-2-1, Nanjing Jiancheng) according to the manufacturer’s protocol. All values were normalized to the total cellular protein content.

### Statistical analysis

Statistical analyses were performed with GraphPad Prism 8.0. Two-tailed unpaired *t*-tests were applied for comparisons between two independent groups. Data are presented as the mean ± standard deviation unless otherwise noted. Statistical significance was determined as follows: ns, not significant; *P < 0.05; **P < 0.01; ***P < 0.001; ****P < 0.0001. All experiments were conducted in triplicate.

## Data Availability

All data generated or analyzed during this study are included in this article and its supplementary material files. Raw sequencing data for RNA sequencing has been deposited in National Center for Biotechnology Information with corresponding accession number: PRJNA1293704. Additional datasetsdata sets and raw data supporting the conclusions of this study are available from the corresponding author upon reasonable request.

## SUPPLEMENTAL MATERIAL

Supplemental figures jvi.00649-25-s0001.pdf Fig. S1 to S8.

ASM does not own the copyrights to Supplemental Material that may be linked to, or accessed through, an article. The authors have granted ASM a non-exclusive, world-wide license to publish the Supplemental Material files. Please contact the corresponding author directly for reuse.
