# Supplementary material for: Membrane protein CRISPR screen identifies RPSA as an essential host factor for porcine epidemic diarrhea virus replication
Source: J Virol. 2025 Jul 30;99(8):e00649-25. doi: 10.1128/jvi.00649-25 (PMC12363229; doi:10.1128/jvi.00649-25)
Supplement: Supplemental figures — Fig. S1 to S8. [file jvi.00649-25-s0001.pdf]

Supplementary Information

Membrane protein CRISPR screen identifies RPSA as an essential host factor for porcine epidemic diarrhea virus replication

Yu Zhao, Guanghao Guo, Yumei Sun, Mengjia Zhang, Gan Yang, Zhongzhu Liu, Yanbin Song, Ahmed H Ghonaim, Ningning Ma, Mengdi Zhang, Anan Jongkaewwattana, Qigai He, Wentao Li

Supplementary Fig 1

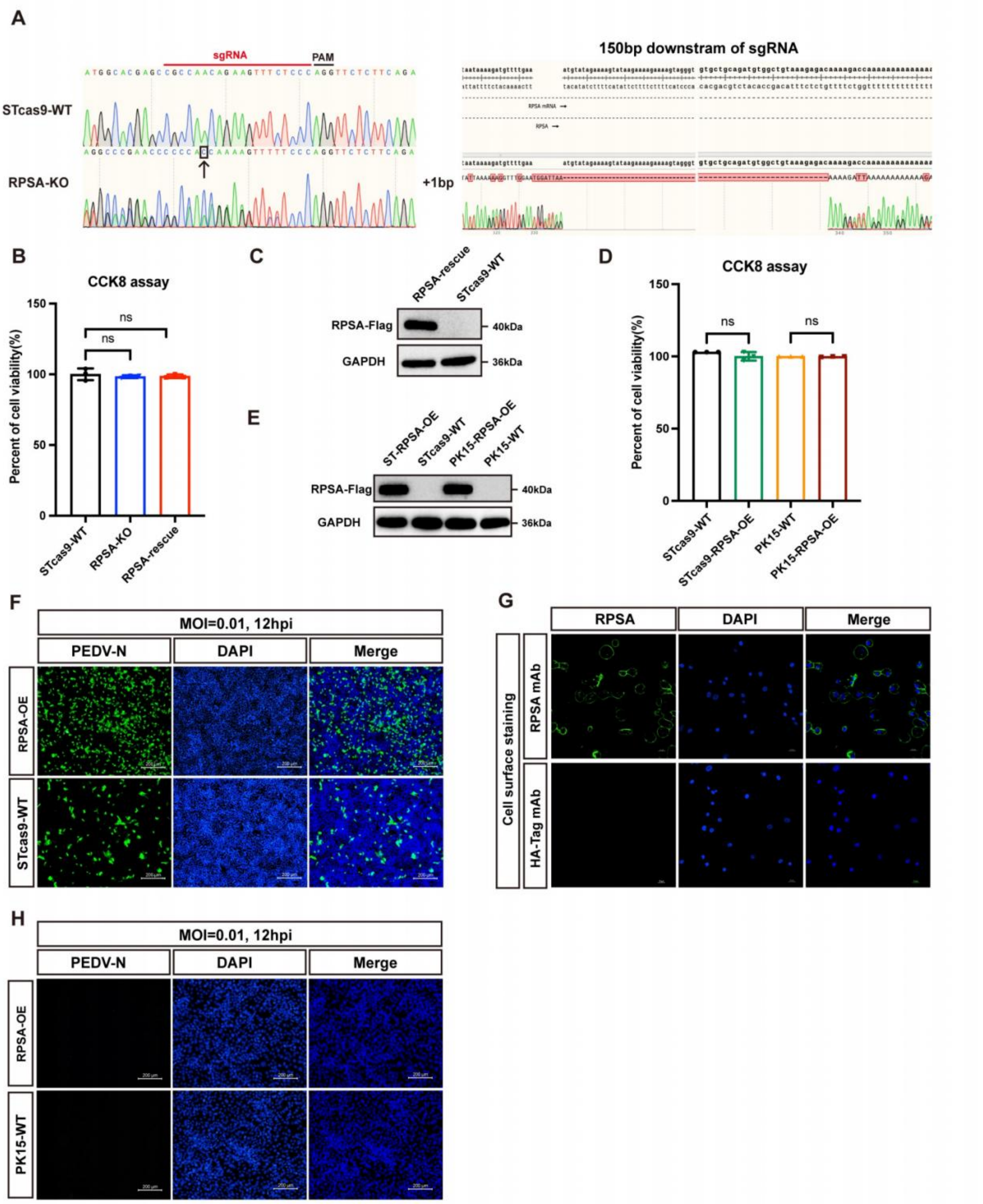

Supplementary Fig 1. RPSA is a host factor required for PEDV infection. (A) Alignment of the nucleic acid sequences of RPSA-KO cells with those of WT cells. The sgRNA-targeted sites and PAM sites are highlighted in red and black, respectively, with the inserted base indicated by the black arrow. (B) Cell proliferation and viability of RPSA-KO, RPSA-rescue, and WT ST cells, as assessed

by CCK-8 assay. (C) Western blot analysis confirming the expression of the Flag-Tagged RPSA protein in the RPSA rescue cell line. (D) CCK-8 assay measuring the proliferation and viability of ST-RPSA-OE, WT-ST, PK15-RPSA-OE, and WT PK-15 cells. ST-RPSA-OE, ST-RPSA-overexpression; PK15-RPSA-OE, PK15-RPSA-overexpression. (E) The protein expression of Flag-Taged RPSA in RPSA-overexpressing cells and WT cells was detected via western blotting with an anti-ST-tag antibody. (F and H) WT cells and RPSA-overexpressing cells were cultured in 24-well plates, and an immunofluorescence assay was used to detect the expression of N protein following infection with PEDV-YN144 (MOI of 0.01) at 12 hpi. Scale bar, 200 μm. (G) Confocal microscopy analysis was used to evaluate the distribution of RPSA located on the surface of ST cells. Scale bar, 10 μm. The means and SDs of the results from three independent experiments are shown. ns, not significant.

Supplementary Fig 2

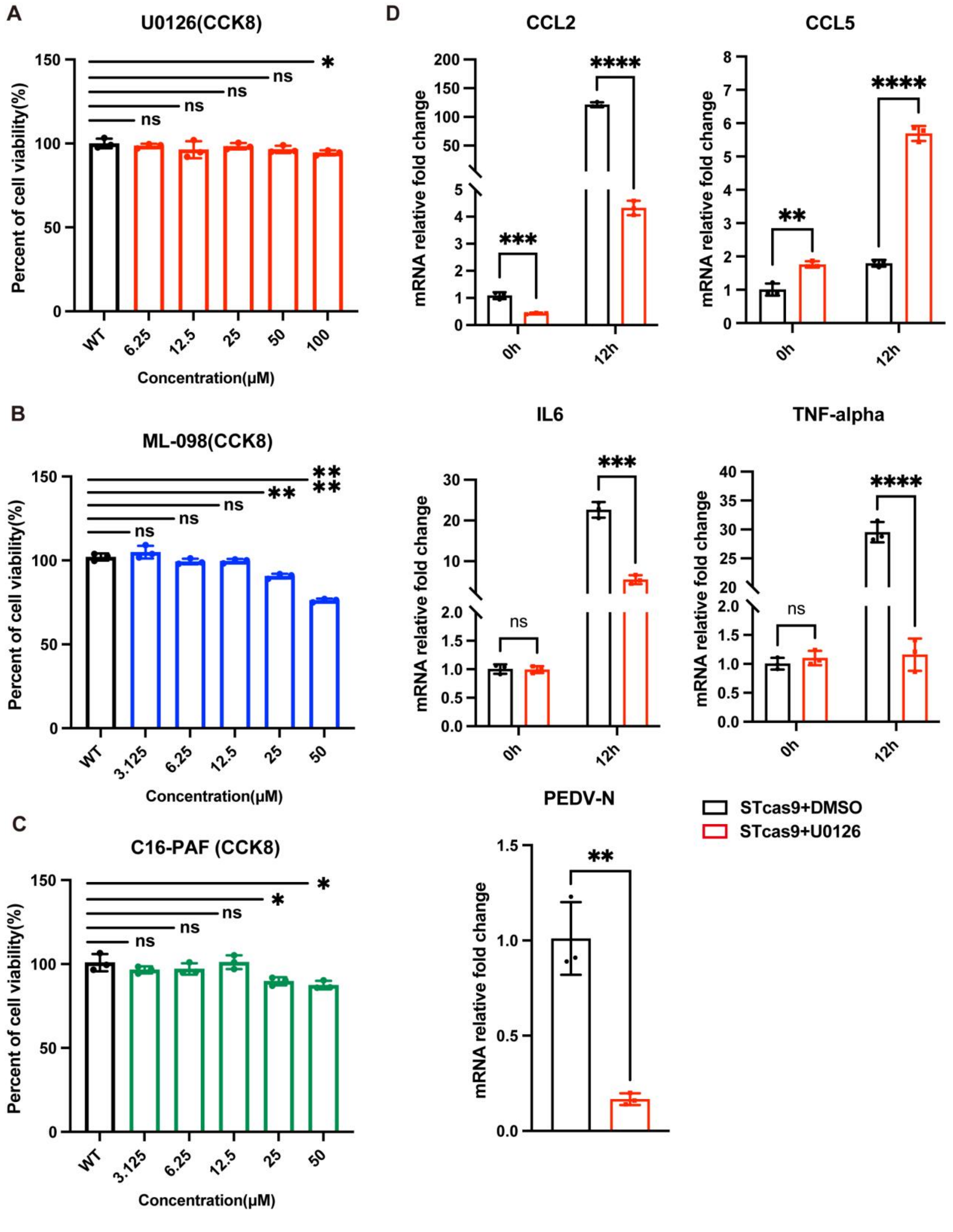

Supplementary Fig 2. Effects of inhibitor or activator treatment on cell viability and inflammatory cytokines. (A) ST cells were treated with or without the inhibitor U0126 for 12 hours, and cell viability was assessed by CCK-8 assay. (B and C) ST cells were treated with or without the activator ML-098 (B) or C16-PAF (C) for 12

hours, and cell proliferation and viability were assessed by CCK-8 assay. (D) ST cells were pretreated with DMSO or U0126 for 2 hours and then infected with PEDV (MOI of 0.01) for 0 or 12 hours. The mRNA expression levels of CCL2, CCL5, IL-6, TNF-α, and PEDV N protein were quantified via RT-qPCR. ns, not significant; * $P < 0.05$; ** $P < 0.01$; *** $P < 0.001$; **** $P < 0.0001$.

Supplementary Fig 3

A

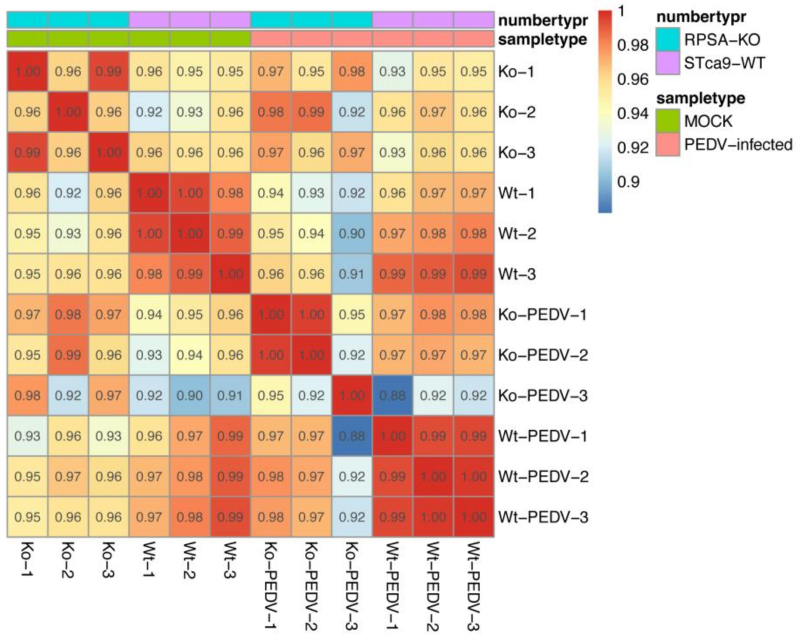

Supplementary Fig 3. Correlations between the RNA-seq samples. (A) Heatmap showing the correlations among the RNA-seq samples. The horizontal and vertical axes represent each sample, with different colors corresponding to varying Pearson correlation coefficients. "KO" refers to uninfected RPSA-KO cells, "WT" refers to uninfected ST cells, "KO-PEDV" refers to PEDV-infected RPSA-KO cells, and "WT-PEDV" refers to PEDV-infected ST cells.

Supplementary Fig 4

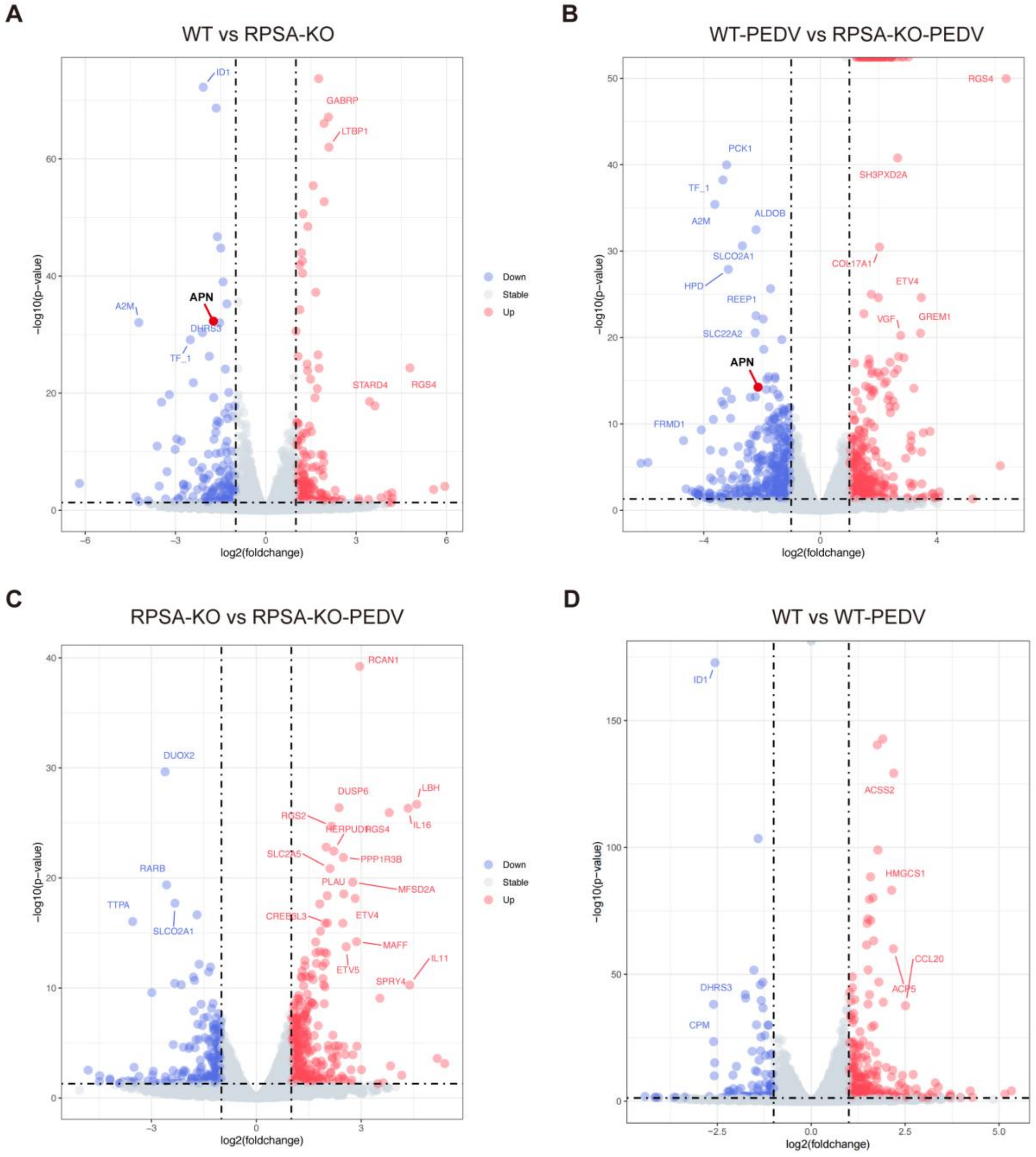

Supplementary Fig 4. Differentially expressed genes across pairwise RNA-seq samples. (A-D) Volcano plots showing DEGs in four pairwise groups: (A) WT vs RPSA-KO, (B) WT-PEDV vs RPSA-KO-PEDV, (C) RPSA-KO vs RPSA-KO-PEDV, and (D) WT vs WT-PEDV. Red dots indicate putative up-regulated genes (p-value < 0.01, log2 fold change > 2), and blue dots indicate putative down-regulated genes (p-value < 0.01, log2 fold change < -2). Notably, the expression of APN was significantly reduced in the absence of RPSA.

Supplementary Fig 5

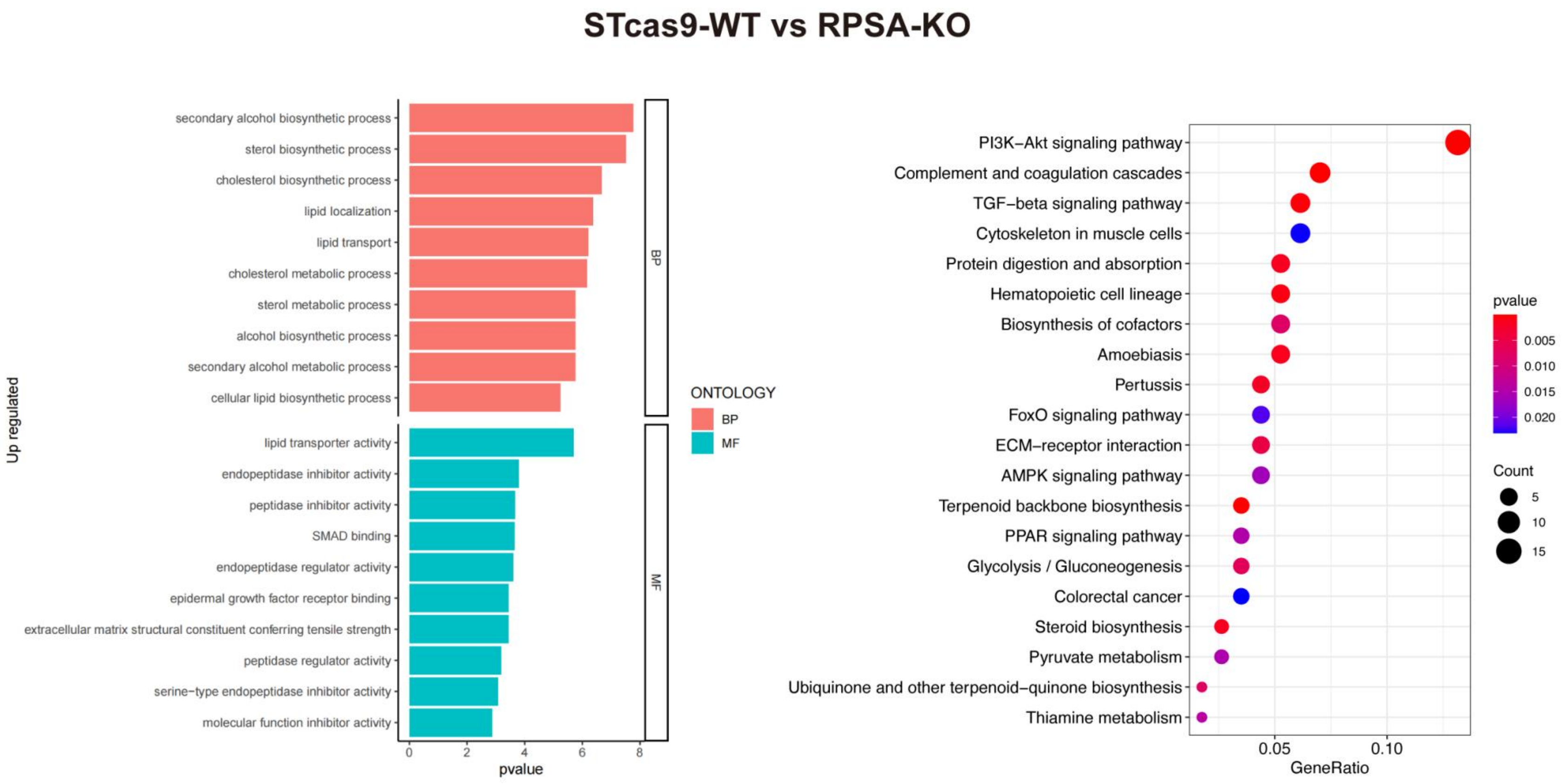

Supplementary Fig 5. KEGG and GO analyses of pairwise RNA-seq samples (WT vs RPSA-KO). The analysis was performed on the basis of the top 50 DEGs, ranked by *p*-value or GeneRatio.

Supplementary Fig 6

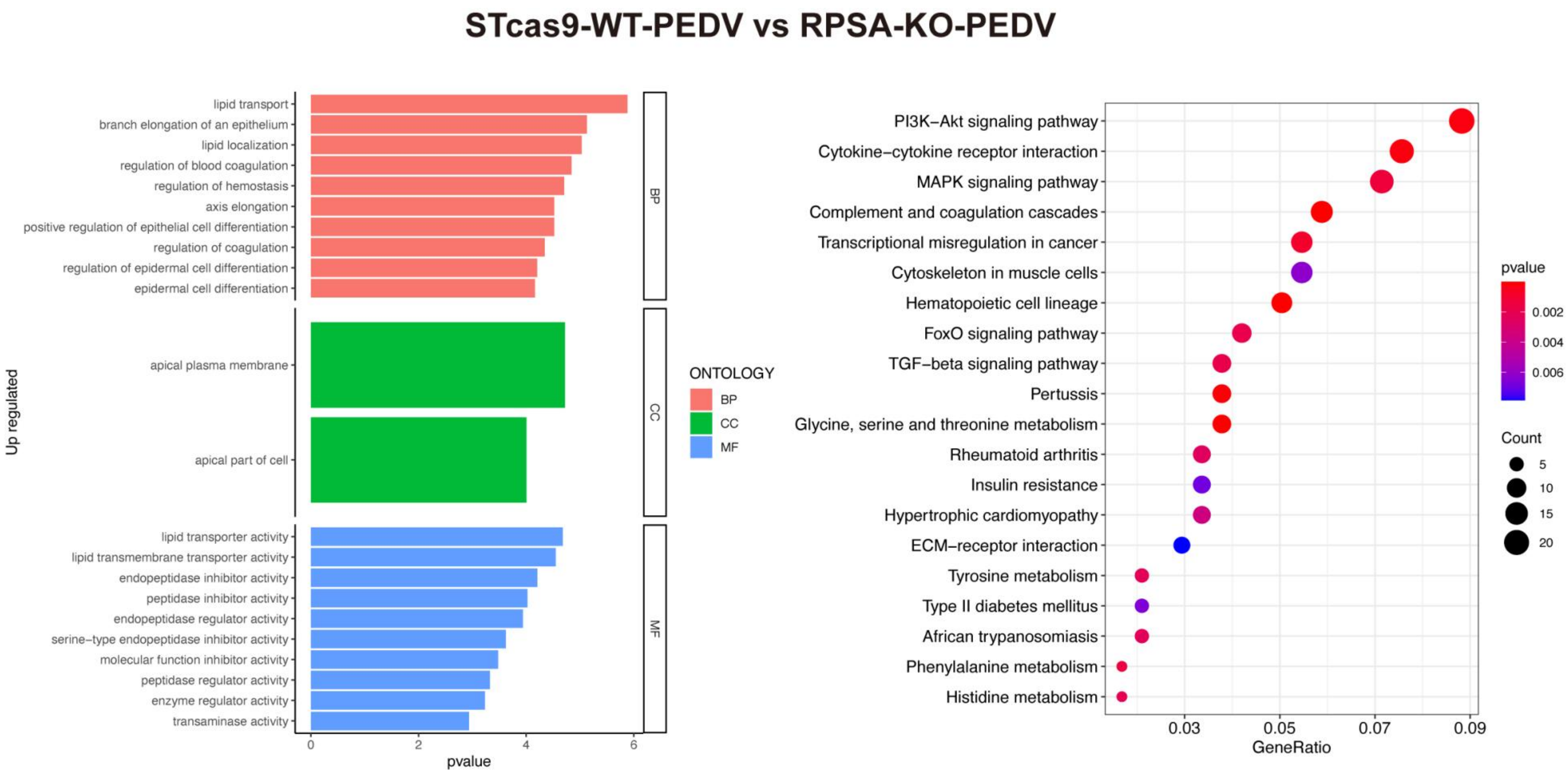

Supplementary Fig 6. KEGG and GO analyses of pairwise RNA-seq samples (WT-PEDV vs RPSA-KO-PEDV). The analysis was performed on the basis of the top 50 DEGs, ranked by *p*-value or GeneRatio.

Supplementary Fig 7

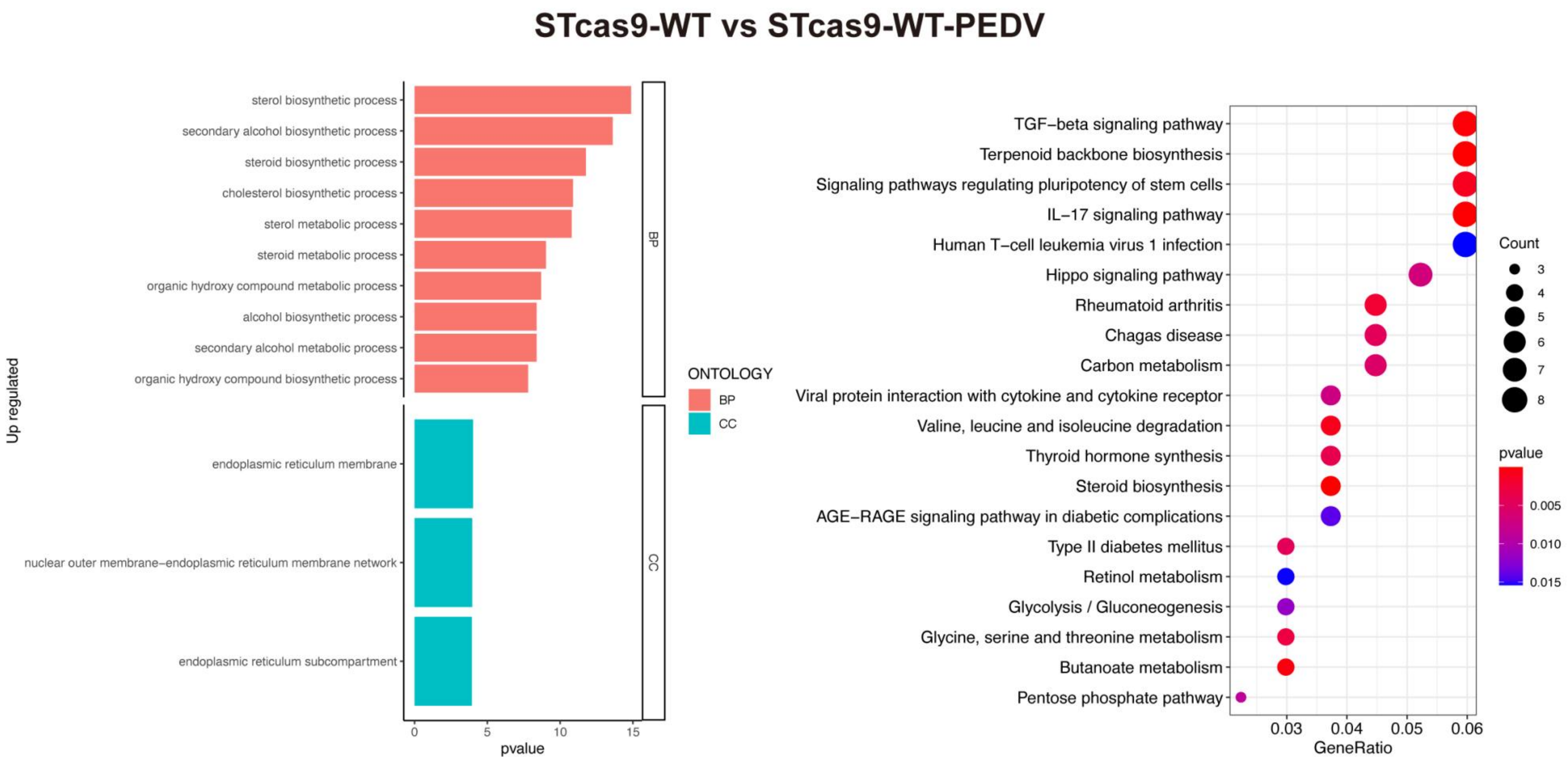

Supplementary Fig 7. KEGG and GO analyses of pairwise RNA-seq samples (WT vs WT-PEDV). The analysis was performed on the basis of the top 50 DEGs, ranked by *p*-value or GeneRatio.

Supplementary Fig 8

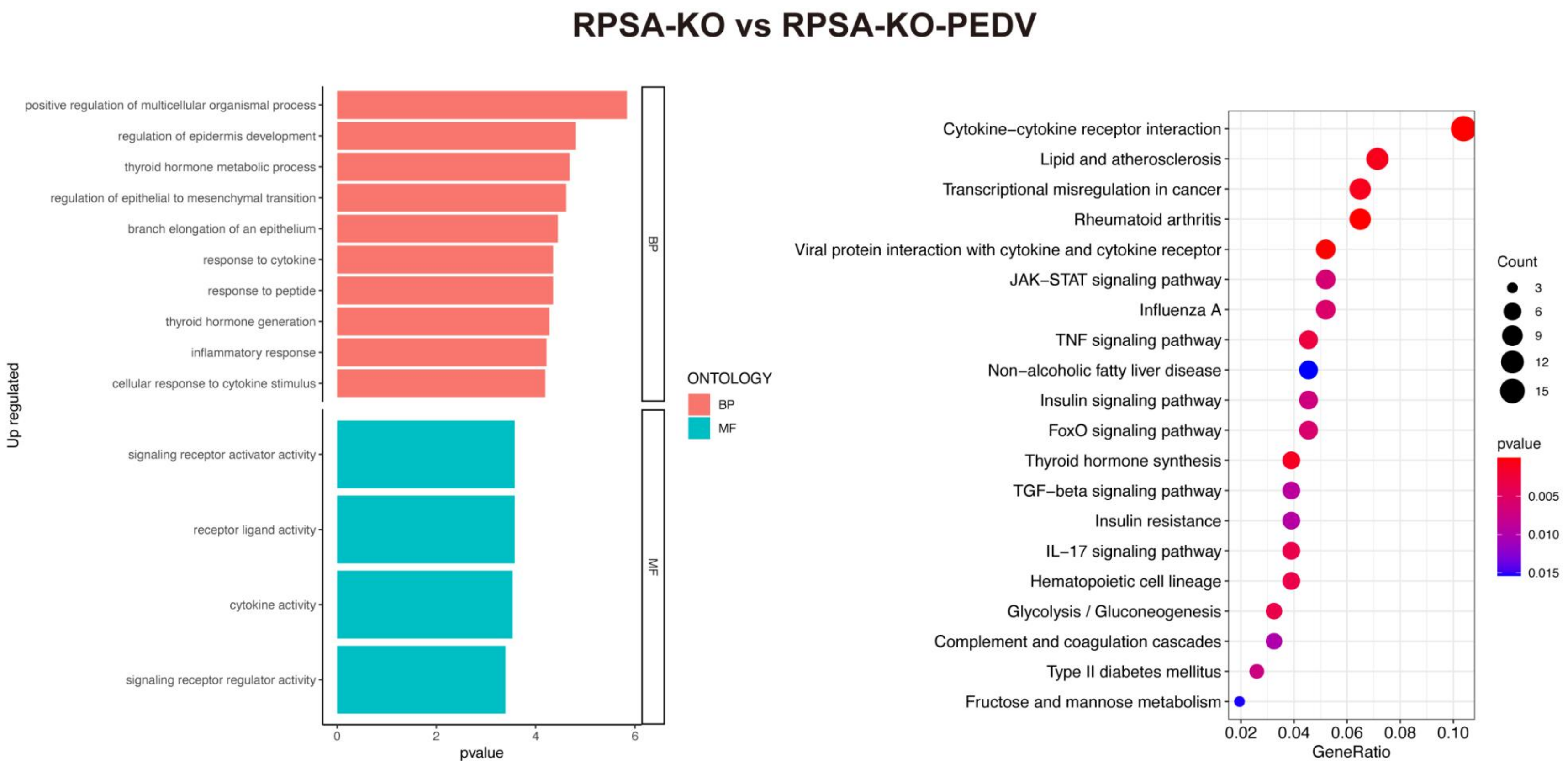

Supplementary Fig 8. KEGG and GO analyses of pairwise RNA-seq samples (RPSA-KO vs RPSA-KO-PEDV). The analysis was performed on the basis of the top 50 DEGs, ranked by *p*-value or GeneRatio.
